# Pre-pandemic contraction, phase-specific rate variation, and site-specific antigenic adaptation shape influenza A(H3N2) evolutionary dynamics in Hubei, China

**DOI:** 10.1128/jvi.00556-26

**Published:** 2026-06-10

**Authors:** Mingwei Peng, Linlin Liu, Xiang Li, Xiao Yu, Qiangling Yin, Xiaolu Zhang, Shi Han, Bin Fang

**Affiliations:** 1 Hubei Provincial Center for Disease Control and Prevention 498598, Wuhan, China; University of Minnesota Twin Cities, Minneapolis, Minnesota, USA

**Keywords:** influenza A(H3N2), COVID-19 pandemic, phylogenetics, evolutionary rate, lineage turnover, selection pressure

## Abstract

Influenza A(H3N2) is a contributor to seasonal epidemics, yet its evolution during and after the COVID-19 pandemic is not fully understood. We analyzed surveillance data and hemagglutinin (HA) and neuraminidase (NA) sequences from Hubei, China, 2017–2024. The epidemic followed regular seasonality before 2020, nearly disappeared during 2020–2022, and resurged with strong peaks after mid-2022. Multiple HA lineages co-circulated pre-pandemic. Several localized reassortment signals were observed before the pandemic, though overall evidence for intra-subtype reassortment remained limited. After resurgence, single dominant clades expanded, genetic diversity dropped, and clade turnover became clear. Bayesian coalescent analyses showed that effective population size had already declined by late 2019, before widespread SARS-CoV-2 transmission and interventions. Relaxed-clock and selection pressure analyses revealed heterogeneous HA substitution rates among lineages, likely reflecting differences in baseline substitution rates and the effects of transmission bottlenecks, rather than detectable shifts in selection intensity. Overall, HA and NA evolution was dominated by purifying selection, with a few sites in antigenic regions showing evidence of positive selection, coinciding with recurrent amino acid changes. Pre-pandemic population contraction, phase-specific rate variation, and site-specific antigenic adaptation shaped lineage turnover and epidemic patterns in Hubei. Monitoring population shifts, lineage-specific rates, and key antigenic substitutions can guide vaccine updates and strengthen preparedness.

**IMPORTANCE:**

The COVID-19 pandemic disrupted influenza activity worldwide, raising questions about its long-term effects on viral evolution. Our analysis of H3N2 in Hubei, China, shows that lineage contraction began before the pandemic and that post-pandemic evolutionary dynamics were shaped primarily by demographic processes, while purifying selection continued to dominate, with adaptive changes restricted to a limited number of antigenic sites. These findings suggest that intrinsic viral processes, not just pandemic restrictions, played a central role in shaping epidemic patterns and highlight the importance of continuous genomic surveillance to anticipate future outbreaks.

## INTRODUCTION

Respiratory viruses cause a heavy global burden, leading to substantial healthcare costs and significant illness and death. Seasonal influenza alone is estimated to cause 290,000–650,000 deaths each year (1). Since the 1968 “Hong Kong flu” pandemic, influenza A(H3N2) has remained a common seasonal subtype (2). It continues to pose a public health concern worldwide, especially among older adults, who experience higher hospitalization and mortality than with other influenza subtypes (3).

Influenza A viruses (Orthomyxoviridae) have an RNA genome with multiple segments, encoding at least eight proteins, including the surface proteins hemagglutinin (HA) and neuraminidase (NA). HA mediates viral attachment and membrane fusion, while NA facilitates viral release. Both proteins are major targets of host immunity (4, 5). The globular head of HA tolerates extensive mutations (6), making it a primary site of antigenic drift. Continuous circulation in humans applies immune pressure on viral surface glycoproteins (7). Together with the error-prone viral polymerase, this leads to ongoing antigen changes, allowing the virus to partially evade immunity from infection or vaccination (8). In addition, A/H3N2 viruses evolve faster than other influenza subtypes (9, 10), with new HA antigenic variants typically emerging every 3–5 years (4). The segmented genome allows occasional reassortment, which can sometimes produce viruses with pandemic potential (11, 12). These features make continuous monitoring and regular vaccine updates necessary.

Previous studies have shown that H3N2 influenza epidemics display distinct seasonality, with peaks occurring in winter–spring in temperate China and during the summer or rainy seasons in subtropical and tropical regions (13, 14). Considerable geographical heterogeneity has also been reported in clade composition and frequent lineage replacement (15). East, South, and Southeast Asia are recognized as important sources of novel variants that frequently spread worldwide (16). Antigenic drift drives recurrent clade replacement, sometimes accompanied by transient co-circulation, before fitter variants rapidly predominate (17). Since 2013, H3N2 viruses within the 3C lineage have undergone rapid clade turnover. Initial dominance of clades 3C.2 and 3C.3 was followed by the emergence of 3C.3a and 3C.2a, the latter defined by key substitutions at antigenic site B that introduced novel glycosylation motifs (18, 19). Subsequently, 3C.3a viruses declined, while 3C.2a became increasingly prevalent (19), and further diversified into multiple subclades (3C.2a1, 3C.2a2/3C.2a3), distinguished by different substitution patterns (17, 20, 21). Before the COVID-19 pandemic, subclade 3C.2a1b, defined by characteristic substitutions such as K92R, R142G, and H311Q, together with its early descendant lineages, gradually became the predominant lineage in global circulation (22, 23).

The COVID-19 pandemic profoundly disrupted influenza circulation, with non-pharmaceutical interventions causing an almost complete interruption of transmission during 2020–2021. In China, B/Victoria viruses predominated in 2021 (24), whereas H3N2 viruses re-emerged and became dominant in 2022, reflecting clade-specific expansions upon resurgence (25). The apparent extinction of the influenza B/Yamagata lineage during the COVID-19 pandemic has raised important questions about whether prolonged circulation gaps could similarly drive H3N2 toward lineage decline or, conversely, facilitate the emergence of new genetic clades (24, 26). H3N2 reemerged with notable epidemics following the pandemic, and recent studies have suggested an accelerated evolutionary rate. However, global root-to-tip regression-based estimates may obscure lineage-specific and temporal heterogeneity in viral evolutionary dynamics (27). Despite these observations, long-term integrated analyses of influenza A(H3N2) remain limited at the regional scale. This is particularly relevant in regions, such as Hubei, which experienced substantial disruption to respiratory virus circulation during the early COVID-19 period. Moreover, the impact of the pandemic on the re-emergence and evolution of H3N2 viruses remains unclear.

To address these gaps, we conducted a longitudinal analysis of influenza A(H3N2) viruses in Hubei Province, China, from 2017 to 2024. By integrating epidemiological and molecular data, we aimed to characterize changes in virus activity and evolutionary dynamics across the pre-, during-, and post-pandemic periods, with particular attention to the roles of population disruption, rate variation, and selective pressures in shaping viral evolution.

## RESULTS

### Temporal dynamics of influenza A(H3N2) in Hubei, 2017–2024

**Fig 1:**
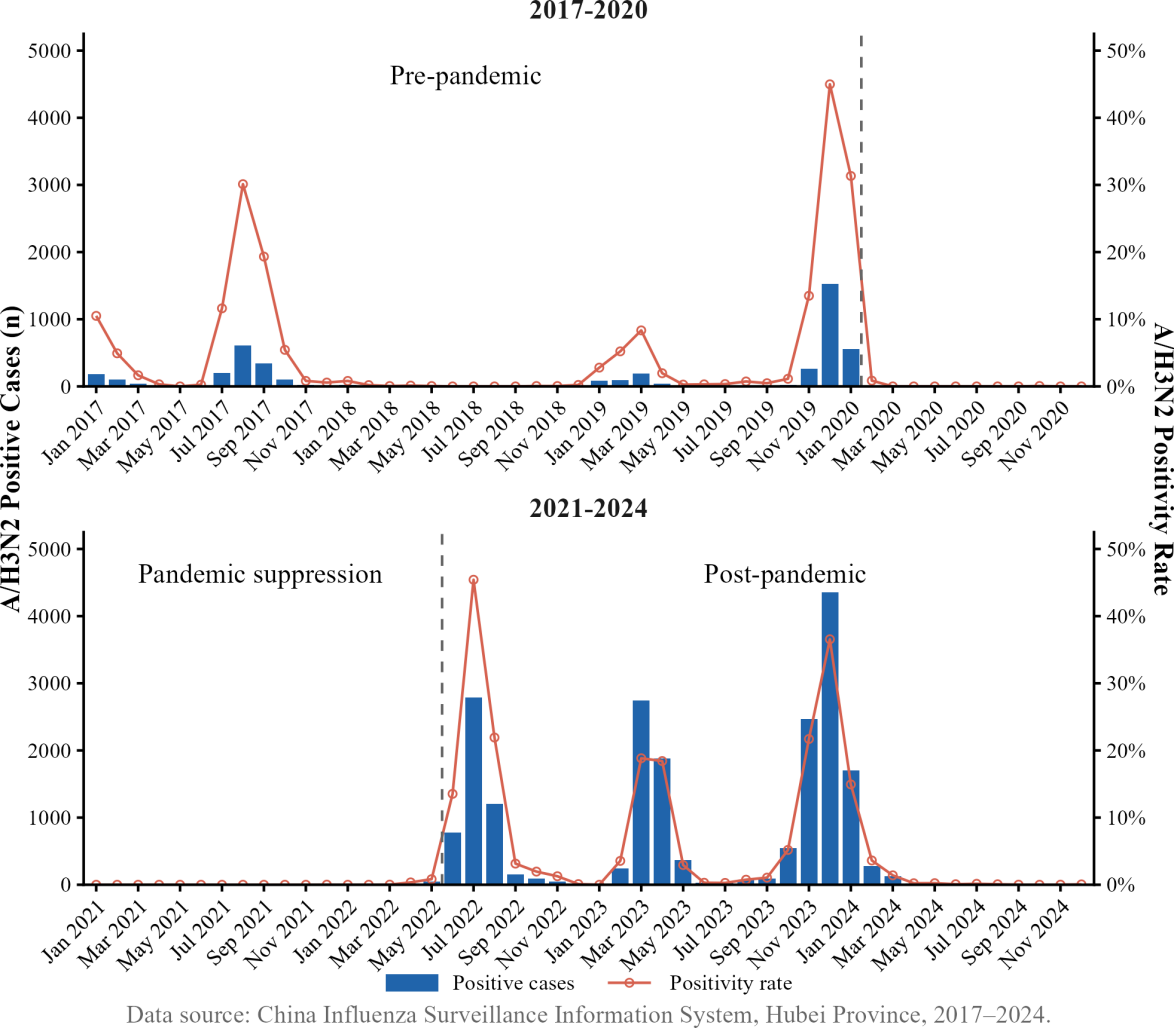
Monthly influenza A(H3N2) activity in Hubei Province, China, 2017–2024. Blue bars show the number of laboratory-confirmed A(H3N2) cases per month, and the red line with circles indicates monthly positivity rates. Vertical dashed lines delineate three epidemiological phases: pre-pandemic (2017–January 2020), pandemic suppression (February 2020–May 2022), and post-pandemic resurgence (June 2022–2024).

Surveillance data from 2017 to 2024 identified 24,616 H3N2 cases among 435,556 respiratory specimens (overall positivity: 5.65%). Monthly positivity patterns revealed three distinct epidemic phases (Fig. 1), as follows.

(i) Pre-pandemic circulation (2017–January 2020): Prior to the COVID-19 pandemic, H3N2 activity in Hubei Province showed recurring winter–spring peaks, with occasional summer increases. A notable summer peak occurred in August 2017 (30.1%), followed by a winter–spring peak during the 2018–2019 influenza season. A major winter peak was subsequently observed in December 2019, with the positivity rate rising to 45.0%. (ii) Pandemic suppression (February 2020–May 2022): H3N2 activity declined sharply in February 2020 and remained at very low levels for 28 months. Surveillance was maintained throughout the COVID-19 pandemic, including over 60,000 specimens tested in 2021, but only sporadic cases were detected (Table S2). (iii) Post-pandemic resurgence (June 2022–2024): H3N2 activity resumed in June 2022, with a marked summer peak in July 2022 reaching the highest positivity rate of the study period (45.4%). This was followed by a winter–spring peak during the 2022–2023 influenza season and a winter peak in 2023, the latter showing the highest monthly case count (4,357 cases) and a positivity rate of 36.6%. Positivity rates declined sharply after December 2023 and remained very low throughout 2024.

Monthly positivity rates differed significantly among the three epidemic phases (Kruskal–Wallis test, *P* < 0.001), supporting temporal heterogeneity in H3N2 activity over the study period.

### Phylogenetic and genetic features of H3N2 viruses in Hubei, 2017–2024

**Fig 2:**
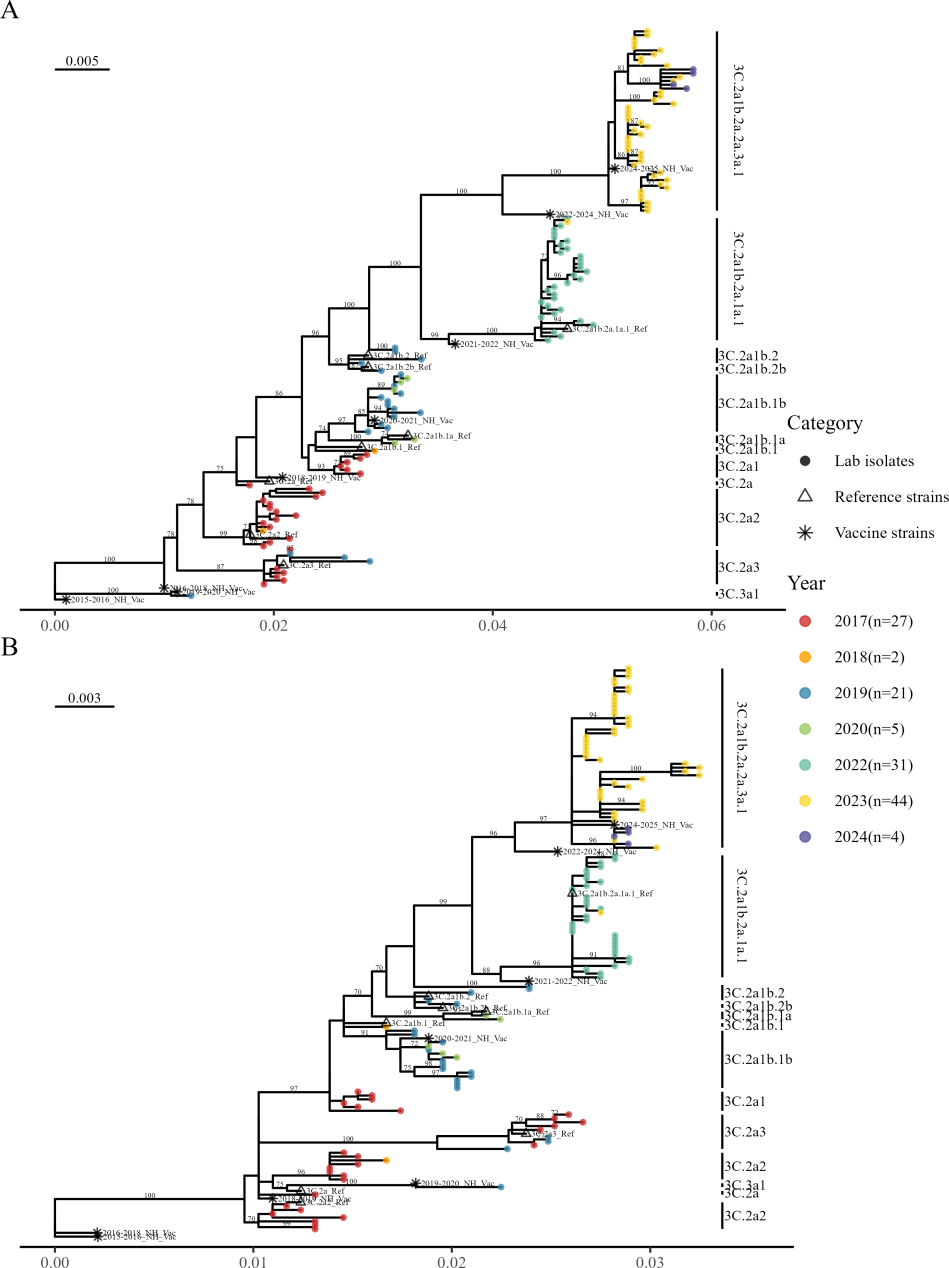
Maximum-likelihood (ML) phylogenetic trees of H3N2 HA and NA gene sequences from Hubei, 2017–2024. (**A**) HA gene; (**B**) NA gene. The scale bar indicates nucleotide substitutions per site. Bootstrap values ≥70% are shown. Tip symbols are colored by sampling year and shaped by sequence category. HA clades are assigned following WHO nomenclature as implemented in Nextclade. In panel B, NA sequences are annotated according to the HA clade of the corresponding isolates to facilitate HA–NA phylogenetic comparison.

Analysis of 134 local isolates together with 16 reference and vaccine strains revealed clear temporal patterns in the HA phylogeny. From 2017 to 2020, multiple clades co-circulated—including 3C.3a1, 3C.2a, 3C.2a1, 3C.2a2, 3C.2a3, and several early-diverging 3C.2a1b lineages—indicating substantial genetic heterogeneity during the pre-pandemic period. After 2020, successive clade turnovers were observed. In 2022, clade 3C.2a1b.2a.1a.1 became predominant, and several isolates had identical nucleotide sequences. In 2023–2024, clade 3C.2a1b.2a.2a.3a.1 dominated the phylogeny and formed a highly homogeneous monophyletic cluster (Fig. 2A).

Compared with HA, the NA phylogeny spans a narrower range of genetic distances based on the *x*-axis branch length scale, indicating a lower level of accumulated genetic divergence, together with partial topological incongruence. In particular, HA clade 3C.2a2 viruses were associated with two distinct NA lineages with variable node support (Fig. 2B). No evidence of intra-segment recombination was detected by RDP4, suggesting that other evolutionary processes may contribute to the observed HA–NA discordance.

### Genetic diversity and vaccine match of H3N2 viruses

**Fig 3:**
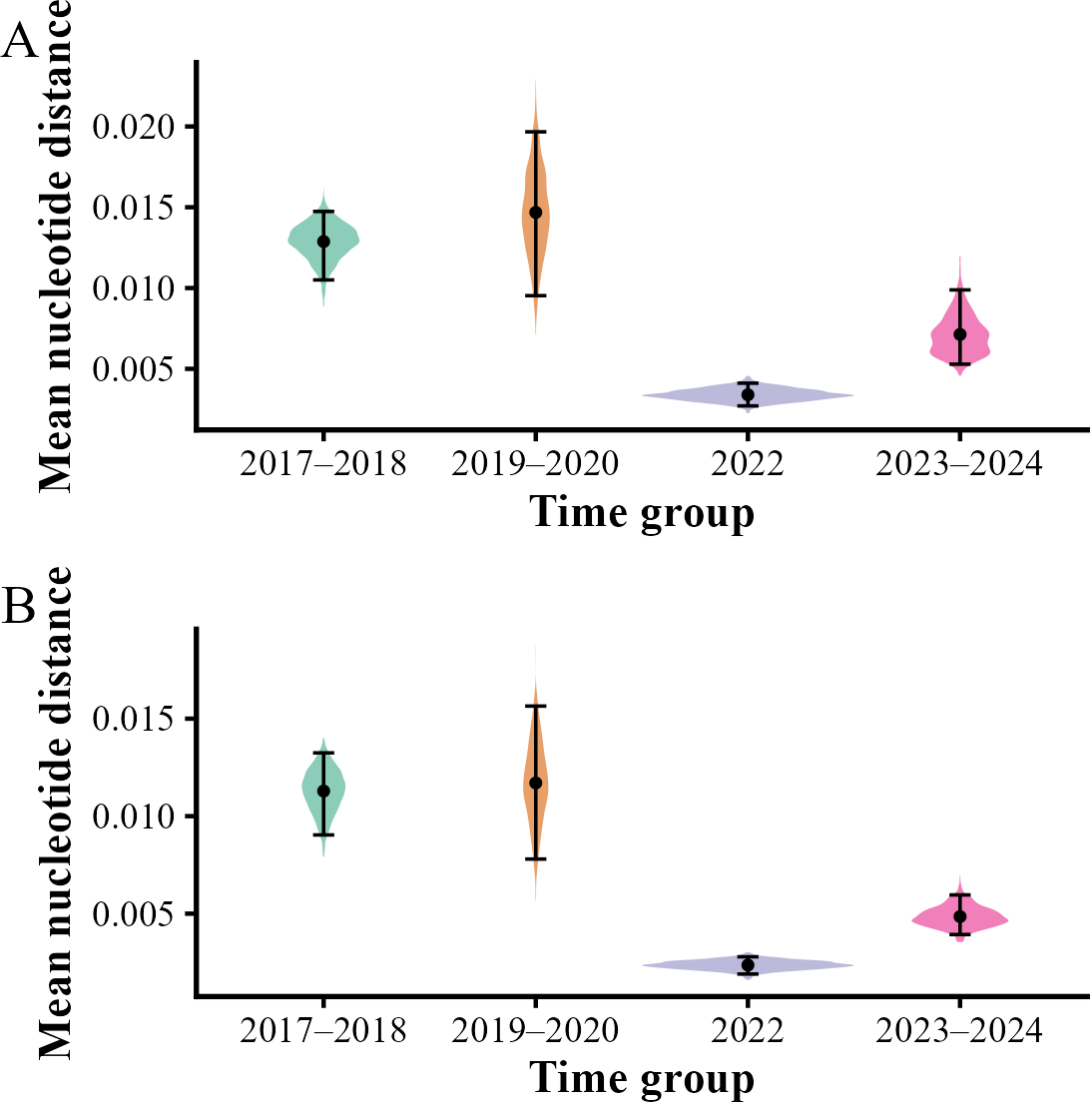
Genetic diversity of H3N2 HA and NA genes. Violin plots show the distribution of bootstrapped mean pairwise nucleotide distances for HA (**A**) and NA (**B**) genes across different epidemic waves. Points indicate the mean distance for each group, and error bars represent 95% confidence intervals. Samples from years with limited isolates were combined with adjacent peak seasons.

Genetic diversity was quantified at the nucleotide level using pairwise distances of the HA and NA segments across different time periods (Fig. 3A and B). For HA, mean pairwise distances were 0.013 and 0.015 in 2017–2018 and 2019–2020, decreasing to 0.003 in 2022 and partially recovering to 0.007 in 2023–2024. Bootstrap pairwise comparisons showed no significant difference between the two pre-2020 periods (95% CI of the difference included 0), whereas all pre- vs post-2020 and post-2020 contrasts yielded differences with 95% CIs excluding 0. A comparable trend was observed for NA, with mean pairwise distances of 0.011, 0.012, 0.002, and 0.005 across periods.

**Fig 4:**
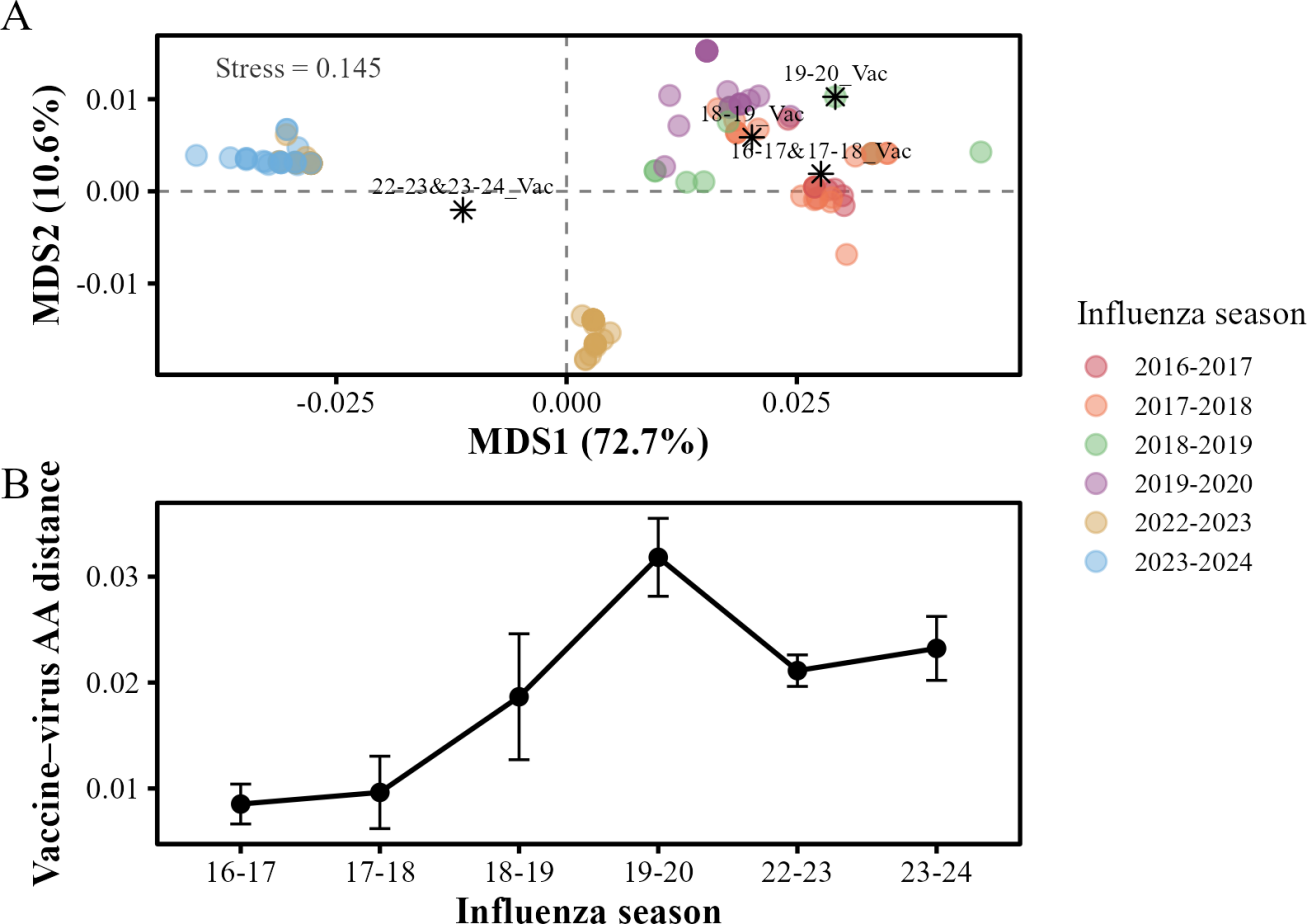
MDS analysis of H3N2 HA proteins and comparison with seasonal vaccine strains. (**A**) HA protein sequences projected by MDS based on pairwise amino acid distances, colored by influenza season. Asterisks indicate recommended vaccine strains. Confidence ellipses were omitted for clarity. (**B**) Mean amino acid distances between circulating viruses and seasonal vaccine strains for each influenza season. Points represent the mean distance, and error bars indicate ± 1 standard deviation.

Vaccine–virus relationships were evaluated at the amino acid level based on HA protein sequences. Multidimensional scaling (MDS) of amino acid distances produced a two-dimensional representation of genetic relationships (stress = 0.145), explaining 69.7% and 10.2% of the variation. Vaccine strains were variably positioned relative to contemporaneous virus clusters (Fig. 4A). Mean HA protein distances ranged from 0.009 ± 0.002 in 2016–2017 to 0.032 ± 0.004 in 2019–2020, with intermediate values in other seasons (Fig. 4B), consistent with vaccine strains being closer to circulating clusters in earlier seasons and becoming more divergent in later seasons, peaking in 2019–2020.

### Temporal signal and model adequacy

Root-to-tip regression showed a clear temporal structure for both HA and NA gene segments of H3N2 viruses sampled in Hubei between 2017 and 2024. The HA gene (Fig. S1A) had an R² of 0.967 and a slope of 0.006, while NA (Fig. S1B) showed a similarly strong correlation (R² = 0.941; slope = 0.002). These results indicate that both data sets contain sufficient temporal signal for molecular-clock inference.

Bayesian model averaging identified a substitution model best fitting both HA and NA, corresponding to a transversion model (TVM)-like variant of the general time reversible (GTR) model (the 123,423 model in Fig. S2A and B). In subsequent BEAST analyses, a GTR parameterization was used in which the A↔G and C↔T substitution rates were fixed as the baseline, while the remaining rates—including the equal A↔T and G↔T rates—were estimated relative to this baseline.

Both strict and relaxed clock models were fitted to the HA and NA data sets. Differences in log marginal likelihoods between clock models were small for both genes (Table S3), indicating no strong preference. However, posterior estimates revealed substantial among-lineage rate heterogeneity for HA (mean ucldStdev = 0.502; 95% HPD: 0.280–0.767). Although NA exhibited lower rate variation (mean ucldStdev = 0.155; 95% HPD: 8.3 × 10⁻⁷–0.446), non-zero heterogeneity could not be excluded; therefore, a relaxed clock was also applied to NA to account for potential rate variation while maintaining analytical consistency between gene segments.

### Time-scaled Bayesian phylogeny of H3N2 viruses

**Fig 5:**
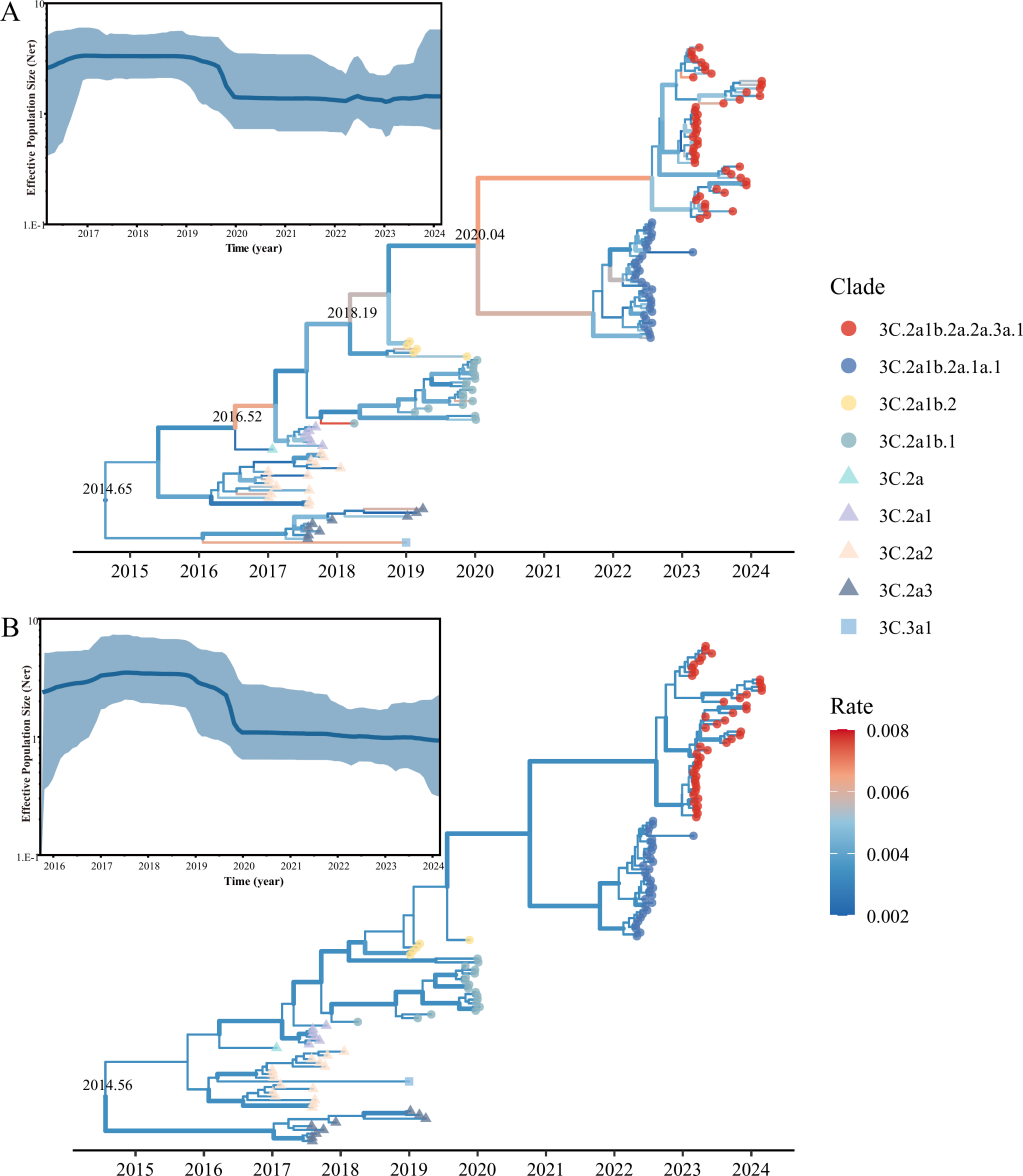
Time-calibrated maximum clade credibility (MCC) trees and demographic history of H3N2 virusesfrom Hubei. (**A**) HA gene; (**B**) NA gene. Branches leading to nodes with posterior support > 95% are shown in bold. Branch colors indicate posterior median substitution rates under the relaxed clock model. Tip points are colored and shaped by clade; closely related subclades are merged for clarity (3C.2a1b.1/1a/1b → 3C.2a1b.1; 3C.2a1b.2/2b → 3C.2a1b.2). Key nodes are labeled with median times to the most recent common ancestor (tMRCA). Bayesian skyline plots (BSPs) based on HA and NA gene sequences are shown as insets in the upper-left corners of the corresponding panels. The solid line represents the median estimate of effective population size (Neτ), and the shaded area represents the 95% highest posterior density (HPD) interval.

The mean substitution rate of the HA gene was estimated at 4.448 × 10⁻³ substitutions/site/year (95% HPD: 3.509–5.458 × 10⁻³), with a time to the most recent common ancestor (tMRCA) of 2014.65 (95% HPD: 2012.09–2015.92) (Fig. 5A). For the NA gene, the mean substitution rate was estimated at 3.409 × 10⁻³ substitutions/site/year (95% HPD: 2.667–4.101 × 10⁻³), while the estimated tMRCA was comparable at 2014.56 (95% HPD: 2013.17–2015.54) (Fig. 5B). HA exhibited a slightly higher posterior mean substitution rate than NA (posterior probability = 0.951), though their 95% HPD intervals remained largely overlapping.

Under the UCLN relaxed molecular clock model, several well-supported nodes (posterior probability >0.95) in the HA maximum clade credibility tree were used as temporal reference points, and descendant branches exhibited relatively elevated posterior median substitution rates. These nodes were dated to 2016.52 (95% HPD: 2015.79–2017.06), 2018.19 (95% HPD: 2017.78–2018.57), and 2020.04 (95% HPD: 2019.28–2020.82) (Fig. 5A). The timing of these lineages coincided with periods of reduced influenza surveillance positivity, including mid-2016, much of 2018, and the period following early 2020 (Fig. 1 and Table S2). Subsequent clade dynamics included the expansion of clades 3C.2a1 and 3C.2a1b.1 after 2016.52, the emergence of clade 3C.2a1b.2 after 2018.19, and the predominance of clades 3C.2a1b.2a.1a.1 and 3C.2a1b.2a.2a.3a.1 after 2020.04. These observations suggest temporal heterogeneity in HA evolutionary rates, with some lineages showing elevated rates during periods of reduced influenza activity.

### Population dynamics and discrete phylogeographic patterns

The temporal dynamics of the effective population size (Neτ) of A(H3N2) viruses in Hubei (2017–2024) were reconstructed using a Bayesian skyline coalescent model under the best-fitting nucleotide substitution and molecular clock model (Fig. 5A and B). The skyline showed three phases: (i) a relatively high and stable plateau from 2017 to early 2019; (ii) a marked reduction in Neτ during mid to late 2019; and (iii) a persistently low, comparatively flat trajectory from January 2020 through 2024. Notably, a sharp contraction in Neτ was observed, with a marked decline beginning in mid-2019 and reaching a low plateau by late 2019, prior to the implementation of COVID-19 non-pharmaceutical interventions in January 2020. Given that sampling intensity broadly mirrored case counts and positivity rates, sampling bias is unlikely to solely explain the observed temporal patterns.

**Fig 6:**
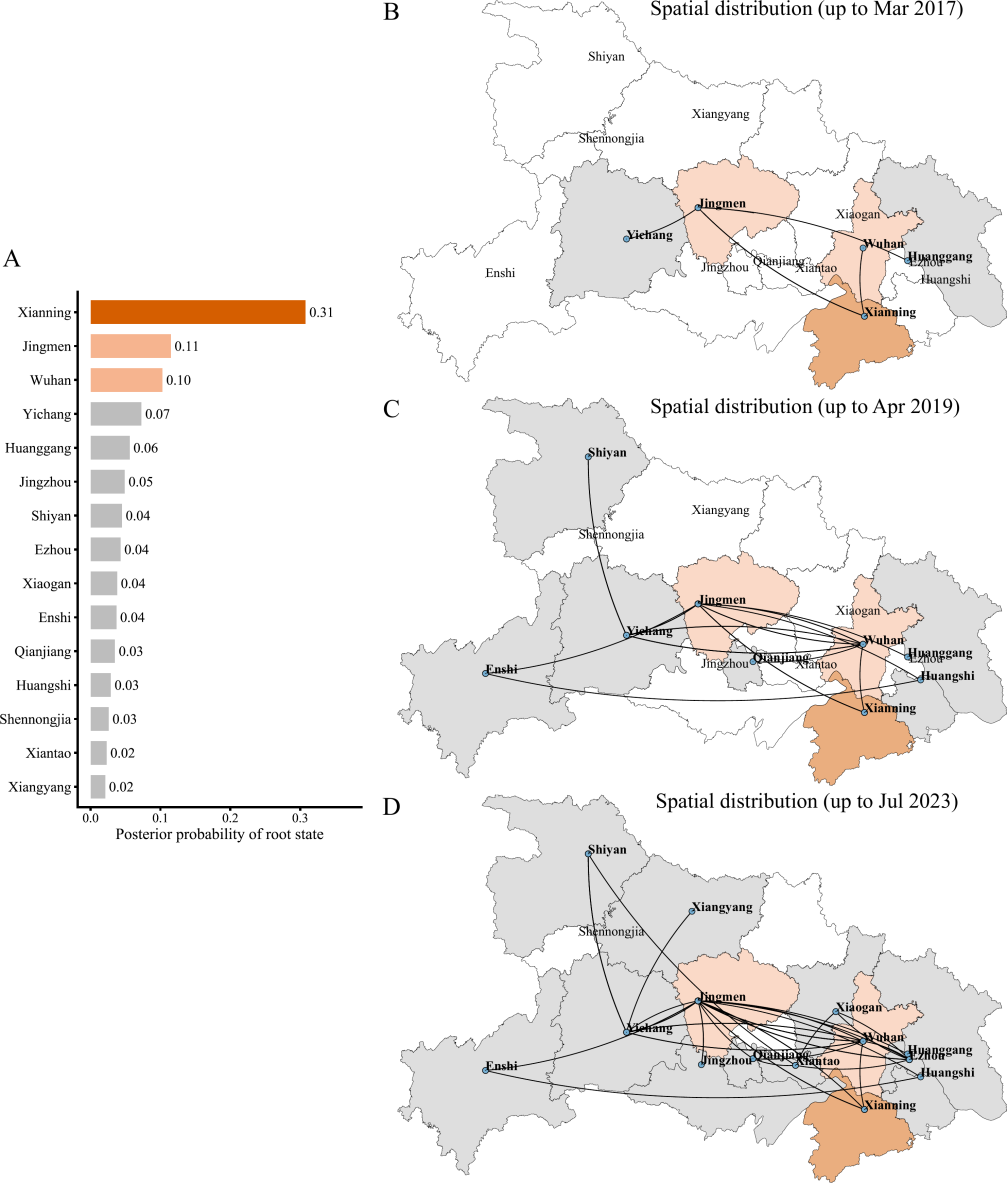
Inferred spatiotemporal diffusion of H3N2 viruses in Hubei (2017–2024). (**A**) Root-state posterior probabilities for sampled prefecture-level cities inferred from the MCC tree. (**B through D**) MCC tree–based diffusion snapshots up to estimated time points (Mar 2017, Apr 2019, and Jul 2023), visualized in SpreaD3. Xianning, Jingmen, and Wuhan (highest root-state posterior probabilities) are highlighted in orange; other cities are shown in gray.

To explore the geographic origin of the sampled lineages, root-state posterior probabilities were estimated using a discrete phylogeographic model. Xianning had the highest posterior probability (0.31), although uncertainty remained high; other cities showed lower support, including Jingmen (0.11) and Wuhan (0.10) (Fig. 6A). MCC tree visualization in SpreaD3 suggested an initial dispersal from Xianning to Jingmen, followed by spread from Jingmen to Yichang and Huanggang. In early 2017, Xianning also contributed to transmission to Wuhan. Subsequently, both Jingmen and Wuhan acted as secondary hubs, mediating transmission to surrounding prefectures and facilitating broader dissemination across Hubei Province (Fig. 6B through D). These patterns reflect inferred diffusion trends rather than statistically supported transmission events.

### Reassortment dynamics revealed by coalescent analysis

To investigate the phylogenetic incongruence observed between HA and NA segments, reassortment dynamics were inferred using a coalescent-based framework implemented in CoalRE within BEAST 2. The estimated mean reassortment rate was 0.166 events/lineage/year (95% HPD: 0.027–0.335), with the credible interval excluding zero.

The MCC network suggested a predominantly tree-like structure with several localized indications of reassortment (Fig. S3). During the pre-pandemic period, multiple putative reassortment events were inferred with relatively high posterior support (>0.85), consistent with ongoing segment exchange among co-circulating lineages. In contrast, only a single low-support event was inferred after 2022, suggesting temporal heterogeneity in reassortment patterns across epidemic periods.

### Selective pressure patterns in HA and NA genes

Site-level selection analyses using the HyPhy package indicated that both the HA and NA genes of A(H3N2) viruses are under strong purifying selection, with overall dN/dS ratios substantially below 1 (ω ≈ 0.26–0.29). The HA gene showed localized evidence of site-specific positive selection, with several sites (e.g., HA positions 160 and 193) consistently identified across multiple methods (Table 1), whereas the NA gene showed little evidence of positive selection, with only weak signals detected by FUBAR and no consistent support across methods.

**TABLE 1:** Site-level selection analysis of the HA gene^*a*^

Method	Sites under diversifying selection (codon; HA pos^*a*^)	No. of sites under purifying selection	Criterion
MEME	147 (131), 160 (144), 176 (160), 209 (193)	–^*b*^	*P* ≤ 0.1
FEL	176 (160), 209 (193), 214 (198)	80	*P* ≤ 0.1
SLAC	176 (160)	25	*P* ≤ 0.1
FUBAR	151 (135), 160 (144), 176 (160), 209 (193)	–	Posterior ≥ 0.9

^*a*^ Codon positions refer to the alignment; HA positions (pos) are based on H3 numbering, excluding the signal peptide.

^*b*^ –, not reported by the method.

Branch-level analysis using aBSREL did not identify any lineages with statistically significant episodic diversifying positive selection in either gene. To further evaluate whether lineages with relatively elevated posterior median substitution rates experienced altered selective pressures, RELAX analyses were conducted using these lineages as test branches. The results showed no significant relaxation or intensification of selection (*K* = 0.82, *P* = 0.364). Consistently, mean dN/dS values were similar between test and reference branches (ω ≈ 0.28 vs 0.26), suggesting no detectable shift in selection intensity among these lineages.

To examine whether branch-specific substitution rates estimated by BEAST were associated with lineage-specific dS, dN, and ω values obtained from CodeML on the same phylogenetic topology, we performed Spearman correlation analyses across corresponding branches. Significant positive correlations were observed for dS (ρ = 0.509, *P* < 0.001), dN (ρ = 0.396, *P* < 0.001), and ω (ρ = 0.328, *P* < 0.001). However, differences among correlation coefficients were not statistically significant based on bootstrap comparison (Table 2).

**TABLE 2:** Spearman correlations between branch-specific substitution rates and lineage-specific dS, dN, and ω, and comparisons among correlation coefficients

Comparison	Spearman ρ	*P* value (ρ)	Δρ	95% CI	*P*-value (Δρ)
Rate–dS	0.509	<0.001	–^*a*^	–	–
Rate–dN	0.396	<0.001	–	–	–
Rate–ω	0.328	<0.001	–	–	–
ρ(rate–ω) vs ρ(rate–dN)	–	–	−0.067	(−0.162, 0.023)	0.139
ρ(rate–ω) vs ρ(rate–dS)	–	–	−0.180	(−0.397, 0.043)	0.107
ρ(rate–dN) vs ρ(rate–dS)	–	–	−0.113	(−0.294, 0.076)	0.225

^*a*^ –, not applicable for the corresponding comparison.

### Temporal dynamics of high-frequency HA substitutions

**Fig 7:**
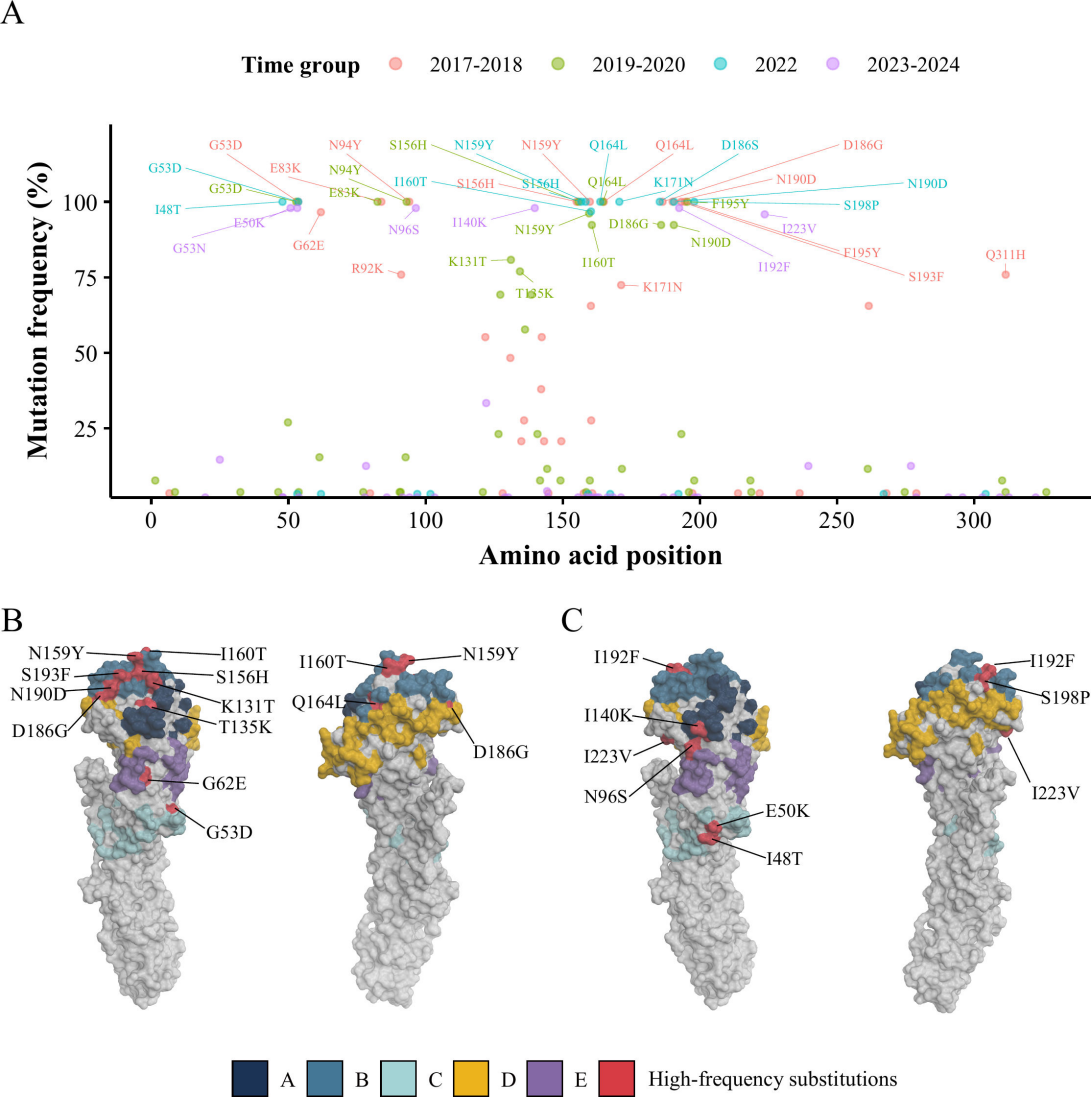
Amino acid substitutions in H3N2 HA (2017–2024). (**A**) HA1 substitutions relative to A/Darwin/6/2021. The x-axis shows amino acid positions, and the *y*-axis shows substitution frequency within each year group. All substitutions are displayed, with ≥70% frequency annotated. Points are colored by year group to illustrate temporal trends. (**B and C**) High-frequency substitutions (≥70%) mapped onto the HA monomer. Antigenic sites A–E are shown in distinct colors, substitutions are highlighted in red, and two orientations are displayed. (**B**) Substitutions prevalent before the pandemic (2017–2020). (**C**) Substitutions emerging after 2020 (2022–2024).

High-frequency substitutions (≥70%) in HA1 relative to A/Darwin/6/2021 were predominantly located in antigenic site B (Fig. 7B). Substitutions including S156H, N159Y, Q164L, and N190D were present prior to the pandemic and persisted across multiple epidemic waves. Temporal replacement patterns were observed at specific sites, such as D186G (2017–2020) being replaced by D186S in 2022, and G53D persisting through 2022 before shifting to G53N in 2023–2024 (Fig. 7A). Other substitutions showed intermittent occurrence across waves, including I160T (2019–2020 and 2022), or were restricted to specific periods, such as G62E and S193F (2017–2018), and K131T and T135K (2019–2020). In the post-2020 period, new high-frequency substitutions emerged, including I48T, E50K, N96S, I140K, I192F, S198P, and I223V (Fig. 7C). Only a limited number of high-frequency substitutions were observed in HA2 (e.g., M193I, I200V), reflecting the dominant role of HA1 in antigenic evolution.

## DISCUSSION

This study comprehensively characterizes the epidemiological and evolutionary dynamics of influenza A(H3N2) viruses in Hubei Province from 2017 to 2024.

Prior to 2020, H3N2 activity showed recurrent winter–spring peaks with occasional summer circulation, consistent with established influenza seasonality in central China (28). Multiple HA clades co-circulated during this period, reflecting substantial genetic heterogeneity, while topological incongruence between HA and NA phylogenies, together with coalescent-based reassortment inference, supports intra-subtype reassortment in H3N2 viruses.

Post-pandemic epidemics were characterized by reduced genetic diversity and successive clade turnover, with single dominant clades expanding and being sequentially replaced against a genetically constrained background (27), accompanied by reduced evidence of reassortment after 2022. H3N2 re-emerged with a summer peak in 2022, followed by the reappearance of typical winter–spring peaks, in contrast to the apparent disappearance of influenza B/Yamagata lineages (26), suggesting a gradual restoration of seasonal influenza activity. HA amino acid divergence between WHO-recommended vaccine strains and circulating viruses varied across seasons, peaking in 2019–2020 and remaining elevated relative to pre-pandemic levels. The unusually intense summer peak in 2022 may reflect increased susceptibility following prolonged suppression of influenza circulation, consistent with “immunity debt” (29).

Phylogeographic analysis suggested Xianning, located at Hubei’s southern gateway bordering southern China, as a possible early source of H3N2, with subsequent spread via Jingmen and Wuhan. This pattern is broadly consistent with south-to-north transmission dynamics in China, where summer influenza activity is often associated with the Lingnan region (30), although limited posterior support suggests cautious interpretation.

Beyond these descriptive patterns, several notable findings emerged from our analyses. Initially, H3N2 Neτ showed a marked decline beginning in mid-2019 and stabilizing at a low plateau by late 2019, preceding both widespread SARS-CoV-2 transmission and COVID-19 non-pharmaceutical interventions (NPIs). While previous studies largely attributed influenza declines to pandemic-related restrictions (31), this earlier contraction may reflect multiple contributing factors, including changes in population immunity driven by influenza vaccination programs (32), and regional or international transmission dynamics (33).

Next, HA substitution rates varied across lineages, with descendant branches from certain internal nodes exhibiting relatively higher posterior median rates under a relaxed molecular clock. While previous studies have reported increased inferred evolutionary rates during the COVID-19 period based on root-to-tip regression analyses (27), our branch-specific relaxed-clock framework revealed heterogeneous rate elevation across multiple lineages and successive epidemic periods, spanning both pre-pandemic and pandemic phases. These high-rate branches frequently coincided with periods of reduced influenza circulation and were followed by the expansion or predominance of specific clades. Given the lack of evidence for shifts in selection intensity (see below), this pattern is more consistent with demographic processes—such as transmission bottlenecks and lineage turnover—than with sustained adaptive evolution, likely reflecting an increased influence of genetic drift, which allows mutations to persist and accumulate along lineages during periods of limited transmission.

Finally, consistent with previous findings in pH1N1 influenza (34), our results indicate that HA evolution in H3N2 is predominantly constrained by purifying selection, as reflected by overall dN/dS ratios well below 1. We found no evidence of episodic diversifying positive selection along individual lineages, and RELAX analyses did not support significant relaxation or intensification of selective pressure in branches with relatively high median substitution rate estimates. Evolutionary rates were positively correlated with dN and dS, and to a slightly lesser extent with ω; these differences were not statistically significant, indicating proportional scaling of synonymous and nonsynonymous substitutions across lineages rather than lineage-specific shifts in selective pressure. This pattern suggests that rate variation is more likely driven by differences in baseline substitution rates and demographic processes rather than by detectable changes in selection intensity, although localized or transient adaptive evolution at specific sites cannot be excluded.

Despite the overall dominance of purifying selection, positively selected sites were identified at a small set of positions, which correspond to well-characterized functional hotspots involved in antigenic evolution, glycosylation changes, and host adaptation. Positions 160 and 193, consistently supported across multiple methods, highlight key roles in immune-driven evolution, with 160 linked to glycosylation at residues 158–160 (35) and 193 located within the receptor-binding region (36). Positions 131–135 and 144 are associated with glycosylation-related antigenic modulation (37), while position 198 is implicated in receptor-binding and host adaptation. These sites overlapped with high-frequency substitutions in our data set, indicating recurrent mutations that tend to reach high frequencies, although not all (e.g., 144) became prevalent, suggesting constraints from viral fitness or epistatic interactions.

In summary, these findings provide new perspectives on the evolutionary trajectories of H3N2 in central China. Our results suggest that pre-epidemic population contractions and phase-specific rate variation, likely reflecting demographic and epidemiological processes, primarily shape these dynamics, with viral evolution largely governed by purifying selection and adaptation limited to a few key sites. Our study is limited by regional sampling and annual case fluctuations, which may have constrained the detection of rare lineages. Moving forward, addressing these gaps through functional assays and integrated global data will be key to guiding public health interventions.

## MATERIALS AND METHODS

### Surveillance data and sample collection

Influenza surveillance data for Hubei Province were obtained from the China Influenza Surveillance Information System (CNISIS) (38). These included RT-qPCR results from respiratory specimens collected at sentinel hospitals and reported by municipal CDCs between January 2017 and December 2024. Influenza A(H3N2)-positive specimens were cultured by municipal CDCs and forwarded to the Hubei Provincial CDC for further analysis. Differences in monthly positivity rates among epidemic phases were assessed using the Kruskal–Wallis test.

### Viral genome sequencing and data processing

Viral RNA was extracted from cultured isolates using the EZ1 Advanced XL automated purification system (QIAGEN, Germany). Full-length gene segments were amplified with primers synthesized by Sangon Biotech (Shanghai, China) based on the 2017 National Influenza Laboratory Technical Guidelines (39), using the SuperScript III One-Step RT-PCR System with Platinum Taq High Fidelity DNA Polymerase (Thermo Fisher Scientific, USA). Sequencing libraries were prepared with the Nextera XT DNA Library Preparation Kit (Illumina, USA) and sequenced on an Illumina MiSeq platform.

Raw sequencing reads were processed in CLC Genomics Workbench 23.0.2 (QIAGEN, Denmark) for quality control, adapter/low-quality base trimming, reference mapping, and consensus sequence generation. The HA and NA gene segments were submitted to GISAID and NCBI GenBank (as described in the Data Availability).

### Phylogenetic reconstruction and clade classification

Evolutionary relationships and clade assignments were determined following standard phylogenetic procedures. Potential recombination events were screened with RDP4 v4.101 before analysis. HA and NA sequences, including reference and WHO vaccine strains, were aligned with MAFFT v7.505 and trimmed with trimAl v1.2rev57 (40). The best-fit nucleotide substitution model (TVM+F+G) was selected by ModelFinder under the Bayesian Information Criterion (BIC) for maximum-likelihood (ML) phylogenetic reconstruction. ML phylogenies were inferred using IQ-TREE v2.4.0 with 1,000 standard bootstrap replicates (41). Clade assignment was performed using Nextclade v3.16.0 (https://clades.nextstrain.org) based on lineage-defining mutations. Trees were visualized and annotated with TreeDataVerse in R v4.5.0.

### Genetic diversity and vaccine match analysis

Genetic diversity among H3N2 isolates was quantified as mean pairwise nucleotide distances calculated in MEGA under the Tamura–Nei model, a standard time-reversible substitution model for distance-based analyses. To maintain statistical robustness while preserving seasonal structure, years with limited sample sizes were merged with adjacent years, resulting in four epidemic wave groups spanning 2017–2018, 2019–2020, 2022, and 2023–2024. Group-level mean distances were estimated using bootstrap resampling of isolates to account for unequal sample sizes, with uncertainty and significance assessed using 95% bootstrap confidence intervals.

Vaccine match was assessed by amino acid distances of the HA protein relative to seasonal vaccine strains using the Jones–Taylor–Thornton model in MEGA. For each influenza season, the mean and standard deviation of isolate-to-vaccine distances were computed to summarize vaccine match. Distance matrices were visualized using multidimensional scaling (MDS) in R to illustrate genetic relationships relative to vaccine strains, with points colored by influenza season.

### Temporal signal assessment and model selection

Temporal signal was evaluated using root-to-tip regression in TempEst v1.5.3 based on a ML tree reconstructed from Hubei isolates, with the root optimized to minimize residual mean squared error. The correlation between sampling dates and root-to-tip genetic distances was quantified by the coefficient of determination (R²) to assess whether the data set contained sufficient temporal signal for reliable molecular clock analysis.

Substitution models for Bayesian analyses were inferred using bModelTest v1.3.3 (42) in BEAST v2.7.7. Markov Chain Monte Carlo (MCMC) convergence was assessed using Tracer v1.7.2 (ESS >200), and posterior model support was visualized using BModelAnalyzer. Molecular clock models were compared using Nested Sampling (NS) v1.2.2 in BEAST v2.7.7 to evaluate strict and uncorrelated lognormal relaxed clocks (UCLN) (43).

### Bayesian evolutionary inference

Bayesian phylogenetic inference was conducted in BEAST v2.7.7 using the substitution and clock models selected previously (44). Rate variation among sites was modeled with a four-category gamma distribution, and a coalescent exponential growth tree prior was applied. MCMC chains were run for 50 million (HA) and 100 million (NA) steps, discarding the first 10% as burn-in. Adequate convergence was confirmed (ESS > 200). The maximum clade credibility (MCC) trees were summarized with median node heights in TreeAnnotator and visualized in R.

### Population dynamics reconstruction and discrete phylogeography

Population dynamics were reconstructed in BEAST v2.7.7 by replacing the coalescent exponential growth tree prior with a Bayesian Skyline Plot coalescent prior, while retaining the same nucleotide substitution model, molecular clock model, and MCMC settings used in the Bayesian evolutionary analyses. Log and tree files were analyzed in Tracer to generate Bayesian Skyline Plots depicting temporal changes in effective population size dynamics.

Discrete phylogeographic analyses were performed in BEAST v2.7.7 using a continuous-time Markov chain (CTMC) discrete trait diffusion model. Sampling locations were defined at the prefecture-level city scale within Hubei Province and encoded as discrete geographic states. The same substitution model, molecular clock model, and coalescent tree prior as in the Bayesian evolutionary analyses were applied. Phylogeographic diffusion patterns were visualized in SpreaD3, with root state probabilities and geographic annotations extracted from the MCC trees (45).

### Reassortment inference

Reassortment was inferred using CoalRE implemented in BEAST v2.7.7 (46). Analyses were performed using the same substitution, clock, and MCMC settings as described above. The posterior distribution of reassortment networks was summarized using a maximum clade credibility–style summary network after burn-in removal. Reassortment rates were estimated from the posterior distribution, and reassortment events and their posterior support were extracted from the same posterior distribution.

### Selection pressure analysis

Site-level selection in the HA and NA genes was analyzed using the HyPhy package (47). Overall nonsynonymous to synonymous substitution rate ratios were estimated to assess overall selection intensity, and multiple site-level methods (MEME, FEL, SLAC, FUBAR) were applied to identify codons under positive selection. To evaluate episodic selection at the lineage level, branch-site analyses were performed using aBSREL, and RELAX was used to test whether predefined lineages exhibited changes in selection intensity relative to reference branches.

To investigate the relationship between evolutionary rates and substitution patterns, branch-specific rates estimated in BEAST were compared with synonymous (dS), nonsynonymous (dN), and dN/dS values inferred from CodeML (48) on the same phylogenetic framework, excluding branches with unreliable estimates (S = number of synonymous sites; S × dS < 1). Spearman rank correlations were calculated separately for rate–dS, rate–dN, and rate–dN/dS associations across corresponding branches, and statistical significance and differences among correlation coefficients were evaluated using non-parametric bootstrap.

### Temporal analysis of HA amino acid substitutions

HA sequences from 2017–2024 were analyzed using Nextclade to identify amino acid substitutions relative to the WHO vaccine strain A/Darwin/6/2021. Substitution frequencies were calculated across predefined temporal groups. All substitutions were visualized using scatter plots, and those with frequencies ≥70% were defined as high-frequency substitutions and annotated. High-frequency substitutions in HA1 were mapped onto the representative H3 HA structure (PDB: 9BDF) using PyMOL (open-source), with antigenic sites indicated.

### Statistical analysis

All statistical analyses were performed using R. Data are presented as the mean ± standard deviation (SD) unless otherwise specified. Statistical significance was assessed using *P*-values or bootstrap confidence intervals depending on the analysis.

## Data Availability

The HA and NA gene sequences generated in this study have been deposited in the GISAID EpiFlu database and NCBI GenBank. Accession numbers are provided in Table S1.

## SUPPLEMENTAL MATERIAL

Supplemental material jvi.00556-26-s0001.docx Fig. S1 to S3; Tables S1 to S3.

ASM does not own the copyrights to Supplemental Material that may be linked to, or accessed through, an article. The authors have granted ASM a non-exclusive, world-wide license to publish the Supplemental Material files. Please contact the corresponding author directly for reuse.
