# Supplementary material for: Pre-pandemic contraction, phase-specific rate variation, and site-specific antigenic adaptation shape influenza A(H3N2) evolutionary dynamics in Hubei, China
Source: J Virol. 2026 Jun 10;100(7):e00556-26. doi: 10.1128/jvi.00556-26 (PMC13386829; doi:10.1128/jvi.00556-26)
Supplement: Supplemental material — Fig. S1 to S3; Tables S1 to S3. [file jvi.00556-26-s0001.docx]

**Supplementary Figures**

**Figure S1.** Root-to-tip regression of H3N2 HA and NA sequences in Hubei, 2017–2024. Root-to-tip regressions were performed on maximum-likelihood trees of Hubei isolates to assess temporal signal. Plots show sampling date versus genetic divergence with regression lines and 95% confidence intervals for (A) HA and (B) NA genes.


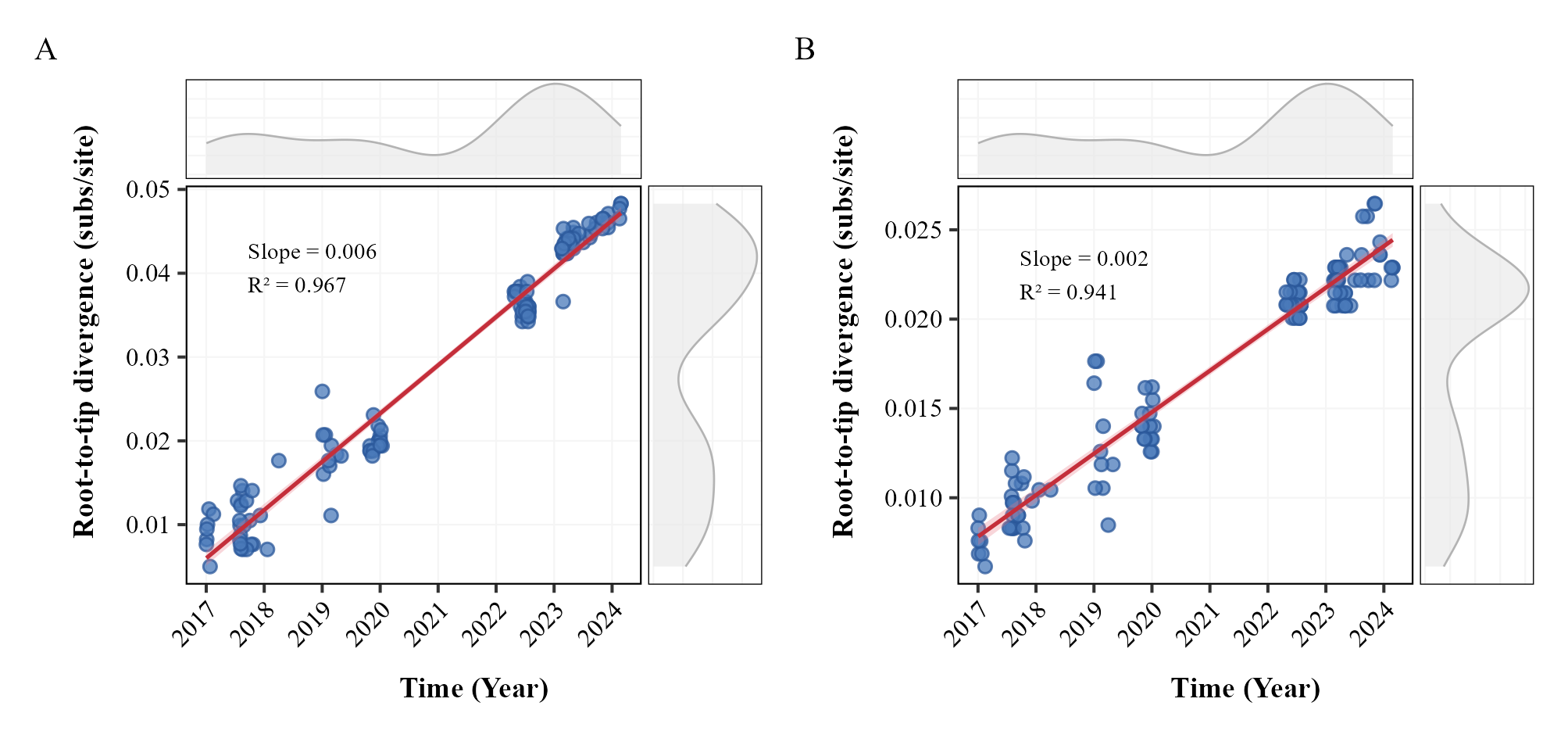


**Figure S2.** Posterior support for nucleotide substitution models. Circle area is proportional to the posterior support. Colors indicate whether the model is included in the 95% credible set (blue) or not (red). The 123423 model showed the highest posterior support for both (A) HA and (B) NA genes.


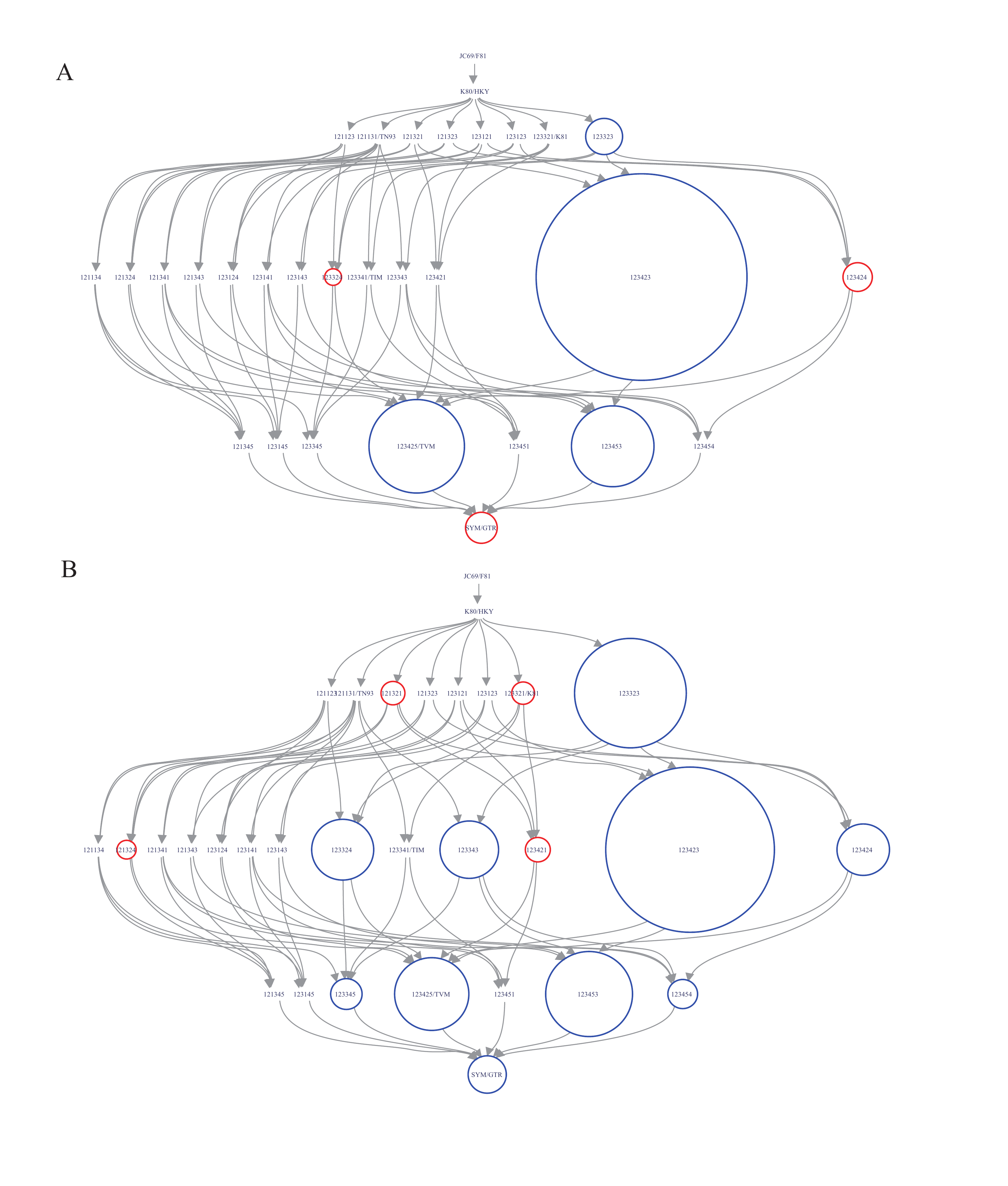


**Figure S3.** Reassortment network inferred using CoalRE. Branches represent phylogenetic relationships, and blue dashed lines indicate inferred HA–NA reassortment events. Four putative reassortment events were identified, including three with high posterior support (posterior probability > 0.85: 0.97, 0.94, and 0.88) and one with low support. Three events were inferred in the pre-pandemic period, while one was inferred after 2022.


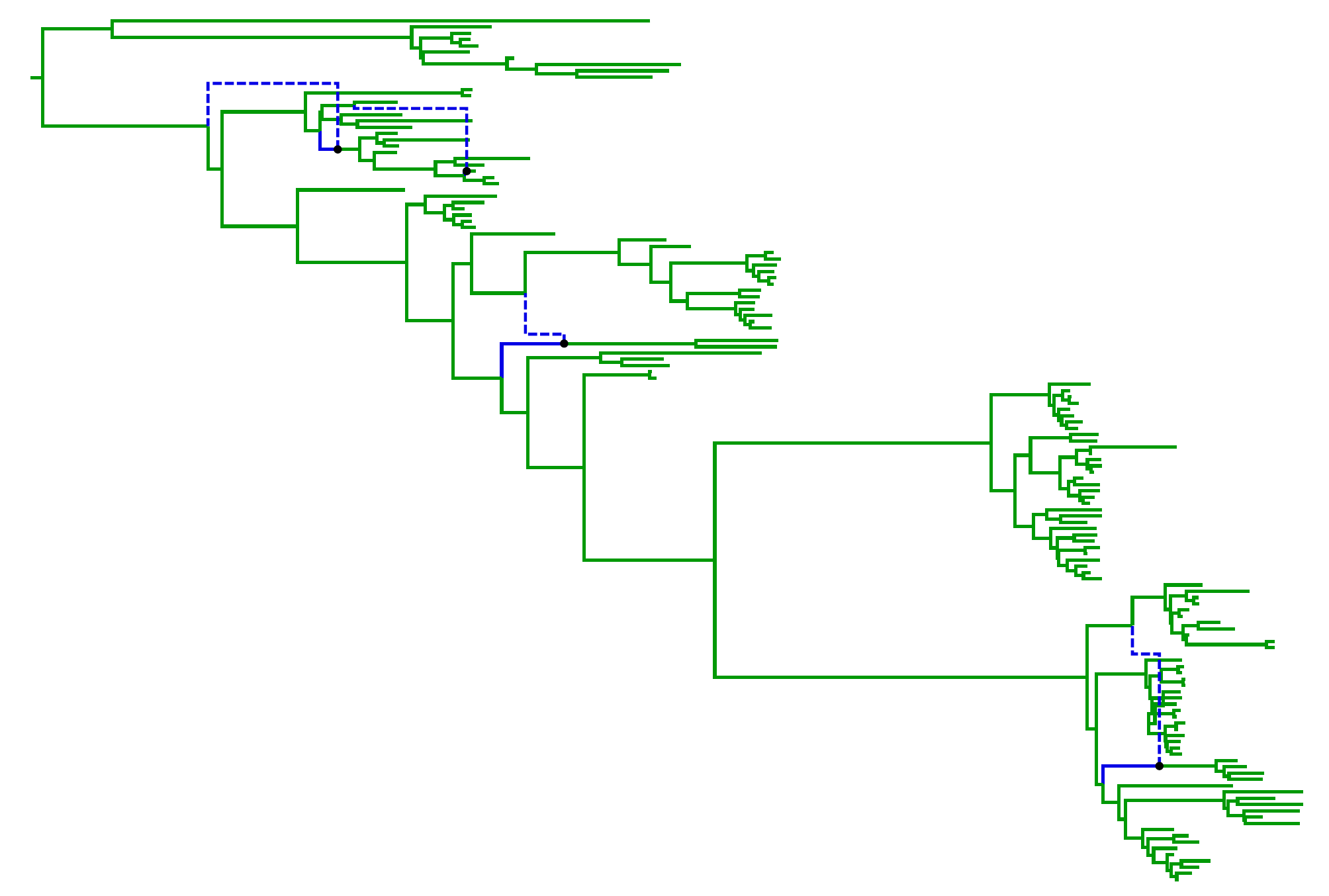


**Supplementary Tables**

**Table S1.** Metadata of H3N2 virus sequences analyzed in this study.

| **Virus name** | **GISAID accession** | **GenBank accession (HA/NA)** | **Collection date** | **Remarks** |
| --- | --- | --- | --- | --- |
| A/Hubei-hanchuan/285/2024 | EPI_ISL_20172888 | PZ368599/PZ368699 | 2024/2/26 | Lab isolates |
| A/Hubei-zhushan/2101/2024 | EPI_ISL_20173034 | PZ368600/PZ368700 | 2024/2/26 | Lab isolates |
| A/Hubei-wuchang/286/2024 | EPI_ISL_20173035 | PZ368601/PZ368701 | 2024/2/17 | Lab isolates |
| A/Hubei-qiaokou/167/2024 | EPI_ISL_20173036 | PZ368602/PZ368702 | 2024/2/17 | Lab isolates |
| A/Hubei-hanchuan/21271/2023 | EPI_ISL_20173037 | PZ368603/PZ368703 | 2023/12/8 | Lab isolates |
| A/Hubei-wujiagang/11868/2023 | EPI_ISL_20173038 | PZ368604/PZ368704 | 2023/12/7 | Lab isolates |
| A/Hubei-huangzhou/11874/2023 | EPI_ISL_20173039 | PZ368605/PZ368705 | 2023/12/7 | Lab isolates |
| A/Hubei-wujiagang/11695FH/2023 | EPI_ISL_20173040 | PZ368606/PZ368706 | 2023/11/2 | Lab isolates |
| A/Hubei-xiangcheng/11801/2023 | EPI_ISL_20173041 | PZ368607/PZ368707 | 2023/11/7 | Lab isolates |
| A/Hubei-wujiagang/11695/2023 | EPI_ISL_20173042 | PZ368608/PZ368708 | 2023/11/2 | Lab isolates |
| A/Hubei-enshi/12353/2023 | EPI_ISL_20173043 | PZ368609/PZ368709 | 2023/9/26 | Lab isolates |
| A/Hubei-zhushan/2749/2023 | EPI_ISL_20173044 | PZ368610/PZ368710 | 2023/9/18 | Lab isolates |
| A/Hubei-shennongjia/21046/2023 | EPI_ISL_20173045 | PZ368611/PZ368711 | 2023/8/22 | Lab isolates |
| A/Hubei-huangshigang/11573/2023 | EPI_ISL_20173046 | PZ368612/PZ368712 | 2023/8/14 | Lab isolates |
| A/Hubei-xianan/11084/2023 | EPI_ISL_20173047 | PZ368613/PZ368713 | 2023/8/8 | Lab isolates |
| A/Hubei-shishou/2752/2023 | EPI_ISL_20173048 | PZ368614/PZ368714 | 2023/7/3 | Lab isolates |
| A/Hubei-jianli/2560/2023 | EPI_ISL_20173049 | PZ368615/PZ368715 | 2023/6/5 | Lab isolates |
| A/Hubei-dongbao/1822/2017 | EPI_ISL_20173050 | PZ368616/PZ368716 | 2017/10/2 | Lab isolates |
| A/Hubei-dongbao/1682/2017 | EPI_ISL_20173051 | PZ368617/PZ368717 | 2017/8/18 | Lab isolates |
| A/Hubei-dongbao/1678/2017 | EPI_ISL_20173052 | PZ368618/PZ368718 | 2017/8/17 | Lab isolates |
| A/Hubei-dongbao/1639/2017 | EPI_ISL_20173053 | PZ368619/PZ368719 | 2017/8/8 | Lab isolates |
| A/Hubei-dongbao/1622/2017 | EPI_ISL_20173054 | PZ368620/PZ368720 | 2017/8/4 | Lab isolates |
| A/Hubei-dongbao/150/2017 | EPI_ISL_20173055 | PZ368621/PZ368721 | 2017/1/18 | Lab isolates |
| A/Hubei-dongbao/122/2017 | EPI_ISL_20173056 | PZ368622/PZ368722 | 2017/1/9 | Lab isolates |
| A/Hubei-dongbao/115/2017 | EPI_ISL_20173057 | PZ368623/PZ368723 | 2017/1/4 | Lab isolates |
| A/Hubei-dongbao/16/2017 | EPI_ISL_20173058 | PZ368624/PZ368724 | 2017/1/2 | Lab isolates |
| A/Hubei-duodao/153/2020 | EPI_ISL_20173059 | PZ368625/PZ368725 | 2020/1/13 | Lab isolates |
| A/Hubei-dongbao/1856/2017 | EPI_ISL_20173060 | PZ368626/PZ368726 | 2017/10/17 | Lab isolates |
| A/Hubei-dongbao/1702/2017 | EPI_ISL_20173061 | PZ368627/PZ368727 | 2017/8/25 | Lab isolates |
| A/Hubei-dongbao/1637/2017 | EPI_ISL_20173062 | PZ368628/PZ368728 | 2017/8/8 | Lab isolates |
| A/Hubei-dongbao/1612/2017 | EPI_ISL_20173063 | PZ368629/PZ368729 | 2017/8/2 | Lab isolates |
| A/Hubei-duodao/1576/2023 | EPI_ISL_20173064 | PZ368630/PZ368730 | 2023/4/5 | Lab isolates |
| A/Hubei-jingshan/2174/2023 | EPI_ISL_20173065 | PZ368631/PZ368731 | 2023/3/1 | Lab isolates |
| A/Hubei-duodao/2196/2023 | EPI_ISL_20173066 | PZ368632/PZ368732 | 2023/5/3 | Lab isolates |
| A/Hubei-duodao/1734/2023 | EPI_ISL_20173067 | PZ368633/PZ368733 | 2023/5/3 | Lab isolates |
| A/Hubei-shayang/2280/2023 | EPI_ISL_20173068 | PZ368634/PZ368734 | 2023/5/13 | Lab isolates |
| A/Hubei-duodao/1711/2023 | EPI_ISL_20173069 | PZ368635/PZ368735 | 2023/5/2 | Lab isolates |
| A/Hubei-dongbao/2110/2023 | EPI_ISL_20173070 | PZ368636/PZ368736 | 2023/5/3 | Lab isolates |
| A/Hubei-duodao/1573/2023 | EPI_ISL_20173071 | PZ368637/PZ368737 | 2023/4/5 | Lab isolates |
| A/Hubei-dongbao/229/2023 | EPI_ISL_20173072 | PZ368638/PZ368738 | 2023/4/13 | Lab isolates |
| A/Hubei-zhongxiang/2273/2023 | EPI_ISL_20173073 | PZ368639/PZ368739 | 2023/4/2 | Lab isolates |
| A/Hubei-jingshan/2282/2023 | EPI_ISL_20173074 | PZ368640/PZ368740 | 2023/3/21 | Lab isolates |
| A/Hubei-duodao/251/2023 | EPI_ISL_20173075 | PZ368641/PZ368741 | 2023/3/16 | Lab isolates |
| A/Hubei-duodao/1358/2023 | EPI_ISL_20173076 | PZ368642/PZ368742 | 2023/3/14 | Lab isolates |
| A/Hubei-jingshan/2209/2023 | EPI_ISL_20173077 | PZ368643/PZ368743 | 2023/3/6 | Lab isolates |
| A/Hubei-duodao/1249/2023 | EPI_ISL_20173078 | PZ368644/PZ368744 | 2023/2/27 | Lab isolates |
| A/Hubei-jingshan/2301/2022 | EPI_ISL_20173079 | PZ368645/PZ368745 | 2022/6/29 | Lab isolates |
| A/Hubei-duodao/1735/2022 | EPI_ISL_20173080 | PZ368646/PZ368746 | 2022/7/18 | Lab isolates |
| A/Hubei-duodao/1772/2022 | EPI_ISL_20173081 | PZ368647/PZ368747 | 2022/7/25 | Lab isolates |
| A/Hubei-jingshan/2344/2022 | EPI_ISL_20173082 | PZ368648/PZ368748 | 2022/7/26 | Lab isolates |
| A/Hubei-duodao/1699/2022 | EPI_ISL_20173083 | PZ368649/PZ368749 | 2022/7/4 | Lab isolates |
| A/Hubei-duodao/130/2020 | EPI_ISL_20173084 | PZ368650/PZ368750 | 2020/1/6 | Lab isolates |
| A/Hubei-dongbao/370/2019 | EPI_ISL_20173086 | PZ368651/PZ368751 | 2019/12/26 | Lab isolates |
| A/Hubei-duodao/11051/2019 | EPI_ISL_20173087 | PZ368652/PZ368752 | 2019/12/23 | Lab isolates |
| A/Hubei-Qianjiang/0423-15/2023 | EPI_ISL_20173088 | PZ368653/PZ368753 | 2023/3/24 | Lab isolates |
| A/Hubei-Qianjiang/0423-14/2023 | EPI_ISL_20173089 | PZ368654/PZ368754 | 2023/3/24 | Lab isolates |
| A/Hubei-Qianjiang/0423-13/2023 | EPI_ISL_20173090 | PZ368655/PZ368755 | 2023/3/24 | Lab isolates |
| A/Hubei-Qianjiang/0423-10/2023 | EPI_ISL_20173091 | PZ368656/PZ368756 | 2023/3/22 | Lab isolates |
| A/Hubei-Qianjiang/0423-07/2023 | EPI_ISL_20173092 | PZ368657/PZ368757 | 2023/3/8 | Lab isolates |
| A/Hubei-Qianjiang/0423-01/2023 | EPI_ISL_20173093 | PZ368658/PZ368758 | 2023/3/3 | Lab isolates |
| A/Hubei-ezhou/1258/2023 | EPI_ISL_20173094 | PZ368659/PZ368759 | 2023/2/20 | Lab isolates |
| A/Hubei-ezhou/1242/2023 | EPI_ISL_20173095 | PZ368660/PZ368760 | 2023/2/20 | Lab isolates |
| A/Hubei-ezhou/20220920-14sample/2022 | EPI_ISL_20173096 | PZ368661/PZ368761 | 2022/7/26 | Lab isolates |
| A/Hubei-ezhou/20220920-09sample/2022 | EPI_ISL_20173097 | PZ368662/PZ368762 | 2022/7/21 | Lab isolates |
| A/Hubei-ezhou/1918/2022 | EPI_ISL_20173098 | PZ368663/PZ368763 | 2022/7/26 | Lab isolates |
| A/Hubei-ezhou/1916/2022 | EPI_ISL_20173099 | PZ368664/PZ368764 | 2022/7/26 | Lab isolates |
| A/Hubei-ezhou/1905/2022 | EPI_ISL_20173100 | PZ368665/PZ368765 | 2022/7/21 | Lab isolates |
| A/Hubei-ezhou/1903/2022 | EPI_ISL_20173101 | PZ368666/PZ368766 | 2022/7/21 | Lab isolates |
| A/Hubei-ezhou/1900/2022 | EPI_ISL_20173102 | PZ368667/PZ368767 | 2022/7/21 | Lab isolates |
| A/Hubei-ezhou/1872/2022 | EPI_ISL_20173103 | PZ368668/PZ368768 | 2022/7/14 | Lab isolates |
| A/Hubei-ezhou/1865/2022 | EPI_ISL_20173104 | PZ368669/PZ368769 | 2022/7/13 | Lab isolates |
| A/Hubei-ezhou/1845/2022 | EPI_ISL_20173105 | PZ368670/PZ368770 | 2022/7/11 | Lab isolates |
| A/Hubei-ezhou/1834/2022 | EPI_ISL_20173106 | PZ368671/PZ368771 | 2022/7/6 | Lab isolates |
| A/Hubei-ezhou/1819/2022 | EPI_ISL_20173107 | PZ368672/PZ368772 | 2022/7/6 | Lab isolates |
| A/Hubei-ezhou/1812/2022 | EPI_ISL_20173108 | PZ368673/PZ368773 | 2022/6/23 | Lab isolates |
| A/Hubei-ezhou/1753/2022 | EPI_ISL_20173109 | PZ368674/PZ368774 | 2022/6/15 | Lab isolates |
| A/Hubei-Qianjiang/0322-13/2023 | EPI_ISL_20173110 | PZ368675/PZ368775 | 2023/2/27 | Lab isolates |
| A/Hubei-Qianjiang/0322-12/2023 | EPI_ISL_20173111 | PZ368676/PZ368776 | 2023/2/27 | Lab isolates |
| A/Hubei-Qianjiang/0322-08/2023 | EPI_ISL_20173112 | PZ368677/PZ368777 | 2023/3/14 | Lab isolates |
| A/Hubei-Qianjiang/0322-07/2023 | EPI_ISL_20173113 | PZ368678/PZ368778 | 2023/3/14 | Lab isolates |
| A/Hubei-Qianjiang/0322-06/2023 | EPI_ISL_20173114 | PZ368679/PZ368779 | 2023/3/14 | Lab isolates |
| A/Hubei-Qianjiang/0322-03/2023 | EPI_ISL_20173115 | PZ368680/PZ368780 | 2023/3/10 | Lab isolates |
| A/Hubei-Qianjiang/0322-02/2023 | EPI_ISL_20173116 | PZ368681/PZ368781 | 2023/3/10 | Lab isolates |
| A/Hubei-Qianjiang/0322-01/2023 | EPI_ISL_20173117 | PZ368682/PZ368782 | 2023/3/10 | Lab isolates |
| A/Hubei-yunmeng/2344/2022 | EPI_ISL_20173118 | PZ368683/PZ368783 | 2022/6/25 | Lab isolates |
| A/Hubei-xiaonan/1777/2022 | EPI_ISL_20173119 | PZ368684/PZ368784 | 2022/6/24 | Lab isolates |
| A/Hubei-hanchuan/5920/2022 | EPI_ISL_20173120 | PZ368685/PZ368785 | 2022/6/15 | Lab isolates |
| A/Hubei-echeng/1745/2022 | EPI_ISL_20173121 | PZ368686/PZ368786 | 2022/6/14 | Lab isolates |
| A/Hubei-xiantao/2651/2022 | EPI_ISL_20173122 | PZ368687/PZ368787 | 2022/6/4 | Lab isolates |
| A/Hubei-danjiangkou/310/2022 | EPI_ISL_20173123 | PZ368688/PZ368788 | 2022/6/1 | Lab isolates |
| A/Hubei-qianjiang/2239/2022 | EPI_ISL_20173124 | PZ368689/PZ368789 | 2022/5/21 | Lab isolates |
| A/Hubei-xiantao/310/2022 | EPI_ISL_20173125 | PZ368690/PZ368790 | 2022/5/19 | Lab isolates |
| A/Hubei-qianjiang/2196/2022 | EPI_ISL_20173126 | PZ368691/PZ368791 | 2022/5/8 | Lab isolates |
| A/Hubei-qianjiang/2185/2022 | EPI_ISL_20173127 | PZ368692/PZ368792 | 2022/5/1 | Lab isolates |
| A/Hubei-qianjiang/2142/2022 | EPI_ISL_20173128 | PZ368693/PZ368793 | 2022/4/27 | Lab isolates |
| A/Hubei-xiantao/2468/2022 | EPI_ISL_20173129 | PZ368694/PZ368794 | 2022/4/26 | Lab isolates |
| A/hubei-wujiagang/19/2020 | EPI_ISL_20173130 | PZ368695/PZ368795 | 2020/1/2 | Lab isolates |
| A/hubei-dongbao/32/2020 | EPI_ISL_20173131 | PZ368696/PZ368796 | 2020/1/2 | Lab isolates |
| A/hubei-chongyang/32/2020 | EPI_ISL_20173132 | PZ368697/PZ368797 | 2020/1/1 | Lab isolates |
| A/hubei-hongshan/312/2019 | EPI_ISL_20173134 | PZ368698/PZ368798 | 2019/12/20 | Lab isolates |
| A/hubei-zhijiang/53/2019 | EPI_ISL_20173135 | PZ368807/PZ368879 | 2019/12/18 | Lab isolates |
| A/hubei-tongcheng/36/2019 | EPI_ISL_20173136 | PZ368808/PZ368880 | 2019/12/23 | Lab isolates |
| A/hubei-maojian/12089/2019 | EPI_ISL_20173137 | PZ368809/PZ368881 | 2019/11/20 | Lab isolates |
| A/hubei-xiangcheng/1936/2019 | EPI_ISL_20173138 | PZ368810/PZ368882 | 2019/11/18 | Lab isolates |
| A/hubei-fancheng/1949/2019 | EPI_ISL_20173139 | PZ368811/PZ368883 | 2019/11/13 | Lab isolates |
| A/hubei-xiling/1766/2019 | EPI_ISL_20173140 | PZ368812/PZ368884 | 2019/10/31 | Lab isolates |
| A/hubei-xiling/1757/2019 | EPI_ISL_20173141 | PZ368813/PZ368885 | 2019/10/29 | Lab isolates |
| A/hubei-xiling/1742/2019 | EPI_ISL_20173143 | PZ368814/PZ368886 | 2019/10/29 | Lab isolates |
| A/hubei-wujiagang/1361/2019 | EPI_ISL_20173144 | PZ368815/PZ368887 | 2019/4/30 | Lab isolates |
| A/hubei-lichuan/510/2019 | EPI_ISL_20173145 | PZ368816/PZ368888 | 2019/4/1 | Lab isolates |
| A/hubei-xiling/1178/2019 | EPI_ISL_20173146 | PZ368817/PZ368889 | 2019/2/28 | Lab isolates |
| A/hubei-enshi/33/2019 | EPI_ISL_20173147 | PZ368818/PZ368890 | 2019/2/26 | Lab isolates |
| A/hubei-maojian/1406/2019 | EPI_ISL_20173148 | PZ368819/PZ368891 | 2019/2/17 | Lab isolates |
| A/hubei-jiangan/1133/2019 | EPI_ISL_20173149 | PZ368820/PZ368892 | 2019/2/11 | Lab isolates |
| A/hubei-jiangan/176/2019 | EPI_ISL_20173150 | PZ368821/PZ368893 | 2019/1/21 | Lab isolates |
| A/hubei-xianfeng/38/2019 | EPI_ISL_20173151 | PZ368822/PZ368894 | 2019/1/9 | Lab isolates |
| A/hubei-jiangan/132/2019 | EPI_ISL_20173152 | PZ368823/PZ368895 | 2019/1/7 | Lab isolates |
| A/hubei-xianan/16/2019 | EPI_ISL_20173153 | PZ368824/PZ368896 | 2019/1/2 | Lab isolates |
| A/hubei-maojian/1153/2018 | EPI_ISL_20173154 | PZ368825/PZ368897 | 2018/1/21 | Lab isolates |
| A/hubei-xiling/1276/2018 | EPI_ISL_20173155 | PZ368826/PZ368898 | 2018/4/3 | Lab isolates |
| A/hubei-hefeng/51/2017 | EPI_ISL_20173156 | PZ368827/PZ368899 | 2017/12/7 | Lab isolates |
| A/hubei-enshi/1887/2017 | EPI_ISL_20173157 | PZ368828/PZ368900 | 2017/10/23 | Lab isolates |
| A/hubei-huangshigang/1894/2017 | EPI_ISL_20173158 | PZ368829/PZ368901 | 2017/10/10 | Lab isolates |
| A/hubei-xiling/1428/2017 | EPI_ISL_20173159 | PZ368830/PZ368902 | 2017/9/12 | Lab isolates |
| A/hubei-qianjiang/2143/2017 | EPI_ISL_20173160 | PZ368831/PZ368903 | 2017/9/11 | Lab isolates |
| A/hubei-jiangan/1640/2017 | EPI_ISL_20173161 | PZ368832/PZ368904 | 2017/8/7 | Lab isolates |
| A/hubei-jiangan/1636/2017 | EPI_ISL_20173162 | PZ368833/PZ368905 | 2017/8/7 | Lab isolates |
| A/hubei-dongbao/1618/2017 | EPI_ISL_20173163 | PZ368834/PZ368906 | 2017/8/4 | Lab isolates |
| A/hubei-jiangan/1619/2017 | EPI_ISL_20173164 | PZ368835/PZ368907 | 2017/7/31 | Lab isolates |
| A/hubei-jiangan/1618/2017 | EPI_ISL_20173165 | PZ368836/PZ368908 | 2017/7/31 | Lab isolates |
| A/hubei-jiangan/1578/2017 | EPI_ISL_278596 | PZ368837/PZ368909 | 2017/7/17 | Lab isolates |
| A/hubei-wujiagang/1144/2017 | EPI_ISL_20173166 | PZ368838/PZ368910 | 2017/2/15 | Lab isolates |
| A/hubei-xianan/172/2017 | EPI_ISL_20173167 | PZ368839/PZ368911 | 2017/1/25 | Lab isolates |
| A/hubei-huangzhou/118/2017 | EPI_ISL_20173168 | PZ368840/PZ368912 | 2017/1/5 | Lab isolates |
| A/Cambodia/e0826360/2020 | EPI_ISL_710475 | / | 2020/7/16 | 2021-2022_NH_Vaccine |
| A/Switzerland/9715293/2013 | EPI_ISL_162149 | / | 2013/12/6 | 2015-2016_NH_Vaccine |
| A/Singapore/INFIMH-16-0019/2016 | EPI_ISL_225834 | / | 2016/6/14 | 2018-2019_NH_Vaccine |
| A/Thailand/8/2022 | EPI_ISL_14991375 | / | 2022/7/11 | 2024-2025_NH_Vaccine |
| A/Hong_Kong/4801/2014 | EPI_ISL_165554 | / | 2014/2/26 | 2016-17/2017-18_NH_Vaccine |
| A/Hong_Kong/2671/2019 | EPI_ISL_377302 | / | 2019/6/17 | 2020-2021_NH_Vaccine |
| A/Darwin/9/2021 | EPI_ISL_2233240 | / | 2021/4/17 | 2022-23/2023-24_NH_Vaccine |
| A/Kansas/14/2017 | EPI_ISL_292575 | / | 2017/12/14 | 2019-2020_NH_Vaccine |
| A/Guangdong-Yuancheng/1587/2017 | EPI_ISL_278602 | / | 2017/7/18 | 3C.2a2_Reference |
| A/Singapore/TT0476/2019 | EPI_ISL_398217 | / | 2019/6/20 | 3C.2a1b.2_Reference |
| A/Hong_Kong/119/2017 | EPI_ISL_253104 | / | 2017/1/4 | 3C.2a_Reference |
| A/Zhejiang-Gongshu/1920/2022 | EPI_ISL_16093965 | / | 2022/9/14 | 3C.2a1b.2a.1a.1_Reference |
| A/Hong_Kong/674/2018 | EPI_ISL_391197 | / | 2018/4/10 | 3C.2a1b.1_Reference |
| A/South_Korea/8831/2019 | EPI_ISL_366590 | / | 2019/3/8 | 3C.2a1b.2b_Reference |
| A/Beijing-Miyun/51/2020 | EPI_ISL_485914 | / | 2020/1/8 | 3C.2a1b.1a_Reference |
| A/Taiwan/1432/2017 | EPI_ISL_304332 | / | 2017/10/25 | 3C.2a3_Reference |

**Table S2.** Monthly H3N2 influenza virus cases and positivity rates in Hubei Province.

| **Year** | **Month** | **Total tests** | **Positive cases** | **Positivity rate (%)** |
| --- | --- | --- | --- | --- |
| 2016 | 1 | 1744 | 5 | 0.29 |
| 2016 | 2 | 1717 | 12 | 0.70 |
| 2016 | 3 | 2672 | 17 | 0.64 |
| 2016 | 4 | 1780 | 8 | 0.45 |
| 2016 | 5 | 1760 | 3 | 0.17 |
| 2016 | 6 | 1523 | 0 | 0.00 |
| 2016 | 7 | 1512 | 3 | 0.20 |
| 2016 | 8 | 1651 | 75 | 4.54 |
| 2016 | 9 | 1538 | 101 | 6.57 |
| 2016 | 10 | 1702 | 168 | 9.87 |
| 2016 | 11 | 2083 | 523 | 25.11 |
| 2016 | 12 | 2022 | 411 | 20.33 |
| 2017 | 1 | 1714 | 180 | 10.50 |
| 2017 | 2 | 2131 | 105 | 4.93 |
| 2017 | 3 | 2297 | 38 | 1.65 |
| 2017 | 4 | 1920 | 6 | 0.31 |
| 2017 | 5 | 1897 | 0 | 0.00 |
| 2017 | 6 | 1575 | 4 | 0.25 |
| 2017 | 7 | 1695 | 197 | 11.62 |
| 2017 | 8 | 2039 | 614 | 30.11 |
| 2017 | 9 | 1785 | 345 | 19.33 |
| 2017 | 10 | 1819 | 99 | 5.44 |
| 2017 | 11 | 2200 | 18 | 0.82 |
| 2017 | 12 | 2445 | 14 | 0.57 |
| 2018 | 1 | 2578 | 21 | 0.81 |
| 2018 | 2 | 1554 | 3 | 0.19 |
| 2018 | 3 | 1796 | 1 | 0.06 |
| 2018 | 4 | 1704 | 2 | 0.12 |
| 2018 | 5 | 1808 | 1 | 0.06 |
| 2018 | 6 | 1568 | 0 | 0.00 |
| 2018 | 7 | 1627 | 0 | 0.00 |
| 2018 | 8 | 1532 | 0 | 0.00 |
| 2018 | 9 | 1432 | 0 | 0.00 |
| 2018 | 10 | 1821 | 1 | 0.05 |
| 2018 | 11 | 1901 | 1 | 0.05 |
| 2018 | 12 | 2269 | 5 | 0.22 |
| 2019 | 1 | 3110 | 87 | 2.80 |
| 2019 | 2 | 1859 | 97 | 5.22 |
| 2019 | 3 | 2242 | 187 | 8.34 |
| 2019 | 4 | 1936 | 38 | 1.96 |
| 2019 | 5 | 1876 | 5 | 0.27 |
| 2019 | 6 | 1631 | 5 | 0.31 |
| 2019 | 7 | 1738 | 6 | 0.35 |
| 2019 | 8 | 1637 | 12 | 0.73 |
| 2019 | 9 | 1681 | 8 | 0.48 |
| 2019 | 10 | 1769 | 20 | 1.13 |
| 2019 | 11 | 1942 | 262 | 13.49 |
| 2019 | 12 | 3388 | 1524 | 44.98 |
| 2020 | 1 | 1768 | 554 | 31.33 |
| 2020 | 2 | 349 | 3 | 0.86 |
| 2020 | 3 | 352 | 0 | 0.00 |
| 2020 | 4 | 464 | 0 | 0.00 |
| 2020 | 5 | 592 | 0 | 0.00 |
| 2020 | 6 | 744 | 0 | 0.00 |
| 2020 | 7 | 1114 | 0 | 0.00 |
| 2020 | 8 | 934 | 0 | 0.00 |
| 2020 | 9 | 1146 | 0 | 0.00 |
| 2020 | 10 | 1685 | 1 | 0.06 |
| 2020 | 11 | 2763 | 0 | 0.00 |
| 2020 | 12 | 3469 | 0 | 0.00 |
| 2021 | 1 | 4528 | 0 | 0.00 |
| 2021 | 2 | 3590 | 0 | 0.00 |
| 2021 | 3 | 5617 | 0 | 0.00 |
| 2021 | 4 | 4960 | 0 | 0.00 |
| 2021 | 5 | 5475 | 0 | 0.00 |
| 2021 | 6 | 5112 | 0 | 0.00 |
| 2021 | 7 | 5245 | 0 | 0.00 |
| 2021 | 8 | 5012 | 0 | 0.00 |
| 2021 | 9 | 5297 | 0 | 0.00 |
| 2021 | 10 | 4831 | 0 | 0.00 |
| 2021 | 11 | 6416 | 0 | 0.00 |
| 2021 | 12 | 6547 | 1 | 0.02 |
| 2022 | 1 | 5474 | 0 | 0.00 |
| 2022 | 2 | 4831 | 0 | 0.00 |
| 2022 | 3 | 5571 | 1 | 0.02 |
| 2022 | 4 | 4838 | 17 | 0.35 |
| 2022 | 5 | 5624 | 47 | 0.84 |
| 2022 | 6 | 5770 | 781 | 13.54 |
| 2022 | 7 | 6132 | 2785 | 45.42 |
| 2022 | 8 | 5474 | 1200 | 21.92 |
| 2022 | 9 | 4763 | 150 | 3.15 |
| 2022 | 10 | 4543 | 89 | 1.96 |
| 2022 | 11 | 4017 | 51 | 1.27 |
| 2022 | 12 | 4144 | 4 | 0.10 |
| 2023 | 1 | 5486 | 0 | 0.00 |
| 2023 | 2 | 6755 | 240 | 3.55 |
| 2023 | 3 | 14590 | 2748 | 18.83 |
| 2023 | 4 | 10229 | 1884 | 18.42 |
| 2023 | 5 | 12662 | 372 | 2.94 |
| 2023 | 6 | 9348 | 28 | 0.30 |
| 2023 | 7 | 9528 | 26 | 0.27 |
| 2023 | 8 | 9559 | 72 | 0.75 |
| 2023 | 9 | 8891 | 94 | 1.06 |
| 2023 | 10 | 10548 | 547 | 5.19 |
| 2023 | 11 | 11390 | 2472 | 21.70 |
| 2023 | 12 | 11920 | 4357 | 36.55 |
| 2024 | 1 | 11413 | 1705 | 14.94 |
| 2024 | 2 | 7790 | 282 | 3.62 |
| 2024 | 3 | 9119 | 128 | 1.40 |
| 2024 | 4 | 8982 | 20 | 0.22 |
| 2024 | 5 | 7649 | 18 | 0.24 |
| 2024 | 6 | 7735 | 6 | 0.08 |
| 2024 | 7 | 9109 | 13 | 0.14 |
| 2024 | 8 | 8140 | 6 | 0.07 |
| 2024 | 9 | 8352 | 1 | 0.01 |
| 2024 | 10 | 8084 | 0 | 0.00 |
| 2024 | 11 | 8066 | 1 | 0.01 |
| 2024 | 12 | 11104 | 6 | 0.05 |

**Table S3.** Assessment of relaxed and strict molecular clock models for HA and NA.

| **Gene** | **Clock model** | **Log marginal likelihood (mean)** | **SD** | **Information** | **Max ESS** |
| --- | --- | --- | --- | --- | --- |
| HA | UCLN | -6103.366560 | 6.120325 | 378.167072 | 444.232030 |
| HA | Strict | -6102.151396 | 5.905763 | 359.421453 | 423.862874 |
| NA | UCLN | -4331.054058 | 5.522329 | 309.448501 | 403.904018 |
| NA | Strict | -4331.388481 | 5.330152 | 307.960955 | 316.458554 |
